# Phage‐Templated Synthesis of Targeted Photoactive 1D‐Thiophene Nanoparticles

**DOI:** 10.1002/smll.202405832

**Published:** 2024-11-05

**Authors:** Paolo Emidio Costantini, Roberto Saporetti, Marika Iencharelli, Soraia Flammini, Maria Montrone, Gennaro Sanità, Vittorio De Felice, Edoardo Jun Mattioli, Mattia Zangoli, Luca Ulfo, Michela Nigro, Tommaso Rossi, Matteo Di Giosia, Emanuela Esposito, Francesca Di Maria, Angela Tino, Claudia Tortiglione, Alberto Danielli, Matteo Calvaresi

**Affiliations:** ^1^ Dipartimento di Farmacia e Biotecnologie Alma Mater Studiorum, Università di Bologna Via Francesco Selmi 3 Bologna 40126 Italy; ^2^ IRCCS Azienda Ospedaliero‐Universitaria di Bologna Bologna 40138 Italy; ^3^ Dipartimento di Chimica “Giacomo Ciamician Alma Mater Studiorum Università di Bologna Via Francesco Selmi, 2 Bologna 40126 Italy; ^4^ Istituto di Scienze Applicate e Sistemi Intelligenti Consiglio Nazionale delle Ricerche Via Campi Flegrei 34 Pozzuoli 80078 Italy; ^5^ Istituto per la Sintesi Organica e la Fotoreattività (ISOF) Consiglio Nazionale delle Ricerche Via Piero Gobetti, 101 Bologna 40129 Italy

**Keywords:** M13 phage, photodynamic therapy, phototheranostic platform, thiophene nanoparticles, virus‐templated synthesis

## Abstract

Thiophene‐based nanoparticles (TNPs) are promising therapeutic and imaging agents. Here, using an innovative phage‐templated synthesis, a strategy able to bypass the current limitations of TNPs in nanomedicine applications is proposed. The phage capsid is decorated with oligothiophene derivatives, transforming the virus in a 1D‐thiophene nanoparticle (1D‐TNP). A precise control of the shape/size of the nanoparticles is obtained exploiting the well‐defined morphology of a refactored filamentous M13 phage, engineered by phage display to selectively recognize the Epidermal Growth Factor Receptor (EGFR). The tropism of the phage is maintained also after the bioconjugation of the thiophene molecules on its capsid. Moreover, the 1D‐TNP proved highly fluorescent and photoactive, generating reactive oxygen species through both type I and type II mechanisms. The phototheranostic properties of this platform are investigated on biosystems presenting increasing complexity levels, from in vitro cancer cells in 2D and 3D architectures, to the in *vivo* tissue‐like model organism *Hydra vulgaris*. The phage‐templated 1D‐TNP showed photocytotoxicity at picomolar concentrations, and the ability to deeply penetrate 3D spheroids and *Hydra* tissues. Collectively the results indicate that phage‐templated synthesis of organic nanoparticles represents a general strategy, exploitable in many diagnostic and therapeutic fields based on targeted imaging and light mediated cell ablation.

## Graphical abstract

The capsid of a refactored M13 phage is decorated with oligothiophene derivatives, transforming the viral structure in a targeted 1D‐thiophene nanoparticle. The unique properties of the bacteriophages (protein‐based structure, possibility to be genetically engineered, tropism for specific cell types) are leveraged on to biofabricate a novel hybrid material, biocompatible, stable, highly photoactive, and receptor‐specific.

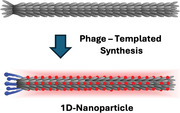

## 1 Introduction

Thiophene‐based nanoparticles (TNPs) are largely used in nanotechnology, particularly in electronics and optoelectronics, as active components of organic light‐emitting diode (OLED), field‐effect transistor (OFET), light‐emitting transistors (LET), lasers, biosensors, chemosensors, and electrochromic devices.^[^ ^1^, ^2^, ^3^, ^4^ ^]^

Because of their excellent electron transport properties, optical properties, soft material nature, stability, solution processability, and water dispersibility, TNPs have recently demonstrated their enormous potential as therapeutic^[^ ^5^, ^6^, ^7^, ^8^, ^9^, ^10^, ^11^, ^12^ ^]^ (photodynamic, PDT; sonodynamic, SDT and photothermal, PTT) and imaging^[^ ^7^, ^9^, ^10^, ^11^, ^13^, ^14^ ^]^ (fluorescent and photoacoustic imaging) agents in nanomedicine.^[^ ^15^ ^]^

Being excellent photo‐transducers TNPs are also considered for the localized and noninvasive photostimulation of cells, such as neurons,^[^ ^16^ ^]^ retinal cells,^[^ ^17^ ^]^ cardiomyocytes^[^ ^18^ ^]^ and even whole animals.^[^ ^19^, ^20^, ^21^, ^22^ ^]^

Two basic synthetic methods are used to address the properties and uses of TNPs:

i) formation of the TNPs in situ during polymer synthesis (bottom‐up approach); ii) preparation of the TNPs after polymer preparation (top‐down approach).^[^ ^23^, ^24^, ^25^ ^]^

Nowadays, post‐polymerization procedures such as mini emulsion and nano‐precipitation/reprecipitation^[^ ^23^, ^24^, ^25^ ^]^ are thought to be the most adaptable ways to create TNPs in water, which is an essential prerequisite for biological applications. Since 3D spherical nanoparticles have the lowest surface‐to‐volume ratio and are thermodynamically advantageous, this is the most common morphology obtained for TNPs.

Despite the many benefits demonstrated in the employment of TNPs in nanomedicine, their use is still hampered by intrinsic limitations: i) it is well known that the shape of a nanoparticle has a crucial role in controlling the cellular uptake and the permeation of physiological barriers,^[^ ^26^ ^]^ but in the case of synthetic methodologies used for TNPs only spherical nanoparticles are commonly obtained,^[^ ^23^, ^24^, ^25^ ^]^ ii) as for all nanoparticles, even if more and more precise synthesis techniques are being developed, intra‐ and inter‐ batch variability issues persist,^[^ ^27^, ^28^ ^]^ iii) TNPs do not have intrinsic target abilities against specific cells.

Virus‐templated synthesis of TNPs may solve all these issues. A growing interest has been shown in biotemplated synthesis of functional nanomaterials for their use in nanotechnology, energy, catalysis, nanomedicine.^[^ ^29^, ^30^ ^]^ This strategy is supported by the special capacity of biological systems to direct the assembly and organization of simple molecules/nanoparticles into complex nanoscale morphologies characterized by new properties and behaviors.^[^ ^31^ ^]^

In particular, i) the genetic control of the synthesis of the virus solves all the issues related to shape/size variability, ii) the size/shape of the virus determines the final morphology of the nanoparticle, iii) viruses intrinsically possess recognition ability of specific cellular receptors, allowing for the development of cellular targeted therapies.

Many viral structures have been used as template for material synthesis and assembly,^[^ ^32^, ^33^ ^]^ including cowpea chlorotic mottle virus (CCMV),^[^ ^34^, ^35^ ^]^ cowpea mosaic virus (CPMV),^[^ ^36^ ^]^ tobacco mosaic virus (TMV)^[^ ^37^ ^]^ and M13 bacteriophage.^[^ ^38^ ^]^

M13 bacteriophage was often employed for the templated synthesis of inorganic nanoparticles.^[^ ^38^, ^39^, ^40^, ^41^, ^42^, ^43^, ^44^, ^45^, ^46^, ^47^, ^48^, ^49^ ^]^ M13 is characterized by a 1D morphology, being ≈6 nm in diameter and 900 nm in length. This aspect ratio confers to the M13 phage an intrinsic capacity of penetration in complex 3D cellular architectures,^[^ ^50^ ^]^ that is, M13 is able to pass across the blood‐brain‐barrier or intestinal barrier.^[^ ^46^, ^51^ ^]^ M13 bacteriophage is very uniform in size and morphology, targeting ligands can be displayed in the tip of the phage, determining its tropism, while its capsid, characterized by a well‐defined surface, can be easily modified.^[^ ^52^ ^]^ For these reasons engineered M13 phages were widely used as delivery systems,^[^ ^52^, ^53^, ^54^, ^55^, ^56^, ^57^, ^58^, ^59^, ^60^, ^61^, ^62^, ^63^ ^]^ in sensors.^[^ ^49^, ^52^, ^61^, ^62^, ^64^, ^65^, ^66^, ^67^, ^68^, ^69^, ^70^, ^71^, ^72^, ^73^, ^74^, ^75^, ^76^ ^]^ or to guide supramolecular assembly.^[^ ^77^, ^78^ ^]^

Here we selected M13 for the templated synthesis of 1D‐thiophene nanoparticles (1D‐TNPs). A refactored M13 phage, engineered by phage display (M13_EGFR_), was used to selectively recognize the epidermal growth factor receptor (EGFR). We covered the surface of the phage with oligothiophene molecules, transforming its viral capsid in a 1D‐TNP, that is, M13_EGFR_(TNP), preserving the original morphology of the virus. After the templated‐synthesis we demonstrated the maintaining of EGFR‐targeted tropism of the phage and the photoactivity of the biohybrid. We tested in vitro the performances of M13_EGFR_(TNP) in photodynamic therapy (PDT) using 2D and 3D cellular models.

Nanosafety and in vivo efficacy of the M13_EGFR_ (TNP) vector in PDT were demonstrated using the tissue like *Hydra vulgaris* polyp as a model system to identify at morphological and molecular levels the pathways elicited by PDT at whole animal level.

## 2 Results and Discussion

**Scheme 1:**
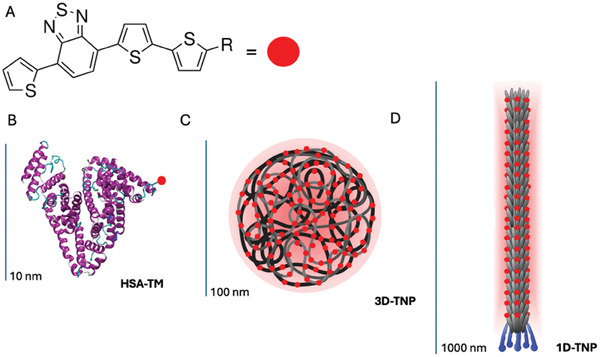
A) Photoactive thiophene molecular scaffold (TM) used for the synthesis of B) human serum albumin ‐ thiophene bioconjugate (HSA‐TM); C) 3D TM‐like polymer thiophene nanoparticle (3D‐TNP) and D) phage templated 1D thiophene nanoparticle (1D‐TNP).

We recently demonstrated the photoactivity of a thiophene molecular scaffold (TM), namely 4‐([2,2′‐bithiophen]‐5‐yl)‐7‐(thiophen‐2‐yl)benzo[c][1,2,5]thiadiazole (**Scheme** **1**A),^[^ ^79^ ^]^ and the possibility of using this hydrophobic molecule in an aqueous physiological environment using its N‐hydroxysuccinimide derivative (NHS‐TM) after conjugation with human serum albumin (HSA‐TM, Scheme 1B).^[^ ^79^ ^]^

### 2.1 Synthesis of 3D Thiophene Nanoparticles (3D‐TNP) by Nanoprecipitation

Thiophene‐based nanoparticles are in principle able to multiply the effect observed with a single molecule. So, spherical 3D thiophene nanoparticles (3D‐TNP) based on the TM core were prepared using a standard two‐step procedure: i) synthesis of a TM‐like polymer, using as repetitive unit that contain a TM derivative, connected by an alkyl linker (Figure S1, Supporting Information); ii) preparation of TM‐like polymer nanoparticles (3D‐TNP) via the reprecipitation method (see details in SI).

The obtained 3D‐TNPs (average size of 95 ± 20 nm) preserve the photophysical properties of the original TM (Figure S2, Supporting Information) and the ability to produce ROS upon irradiation (Figure S3, Supporting Information). However, in vitro cellular results (Figure S4, Supporting Information) showed that the synthesized 3D‐TNPs are extremely less efficient in PDT than the HSA‐TM bioconjugates. So, an alternative procedure for the synthesis of TNPs was developed.

### 2.2 Phage‐Templated Synthesis of 1D Thiophene Nanoparticles – M13_EGFR_(TNP)

**Figure 1:**
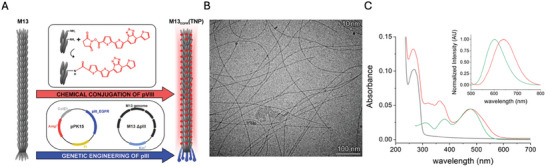
A) Development of phage‐derived 1‐D thiophene nanoparticles M13_EGFR_(TNP). M13 was first genetically engineered to target EGFR‐positive cells (M13_EGFR_) and then chemically conjugated with oligothiophene derivatives. B) A representative cryo‐TEM image of M13_EGFR_(TNP) in PBS 1x; C) Spectroscopic characterization of the M13_EGFR_(TNP) hybrid in PBS 1x. UV–vis spectra of M13_EGFR_ (black line), oligothiophene derivative (green line), and purified M13_EGFR_(TNP) (red line). The inset shows the fluorescence spectra of the oligothiophene derivative (green line) and M13_EGFR_(TNP) (red line).

By using HSA, on average 1.5 TM molecules were conjugated to a single protein.^[^ ^79^ ^]^ A virus, whose capsid offers hundreds of available sites for bioconjugation, can be employed as a template for the synthesis of a 1D‐TNP (Scheme 1C). We used an engineered M13 phage, genetically refactored to display on the phage tip a peptide (SYPIPDT) able to bind EGFR.^[^ ^80^, ^81^ ^]^ Using an orthogonal approach (nanoarchitectonics),^[^ ^82^ ^]^the surface of the capsid was chemically functionalized (**Figure** **1**A). The oligothiophene molecules were conjugated via cross‐coupling reaction between the succinimidyl ester (NHS) moiety of the oligothiophene derivatives (and the amino acid amine groups of M13_EGFR_ capsomers (Figure 1A).

The synthetic process generated monodispersed, uniformly sized 1D‐TNP (6.3 ± 0.45 nm width), fully maintaining the morphological characteristics of the template phage (Figure 1B). The genetic control of the virus assembly ensures a stringent reproducibility of the size/shape of the capsid template, reducing the common problem related to intra‐batch and batch to batch variability in the synthesis of nanoparticles. In fact, the diameter of the 1D nanoparticle is strictly maintained (Figure S7, Supporting Information) within the same batch, or in different batches. Even if the use of absorption intensity to quantify the exact number of oligothiophene molecules contained in a nanoparticle is not rigorous (many approximations must be taken in account), considering the initial M13_EGFR_ concentration and the molar extinction coefficient of the oligothiophene derivative, we can estimate an average number of 416 TM molecules attached to the capsid of the M13_EGFR_ phage in a M13_EGFR_(TNP) (Figure 1C). The absorption band of M13_EGFR_(TNP), centered at 478 nm became broader when compared to the oligothiophene molecules in solution. This broadening hints at a complex interaction between the anchored molecules and the capsid surface, resembling the typical behavior of the oligothiophene molecules in the solid state. On the opposite, the absorption peak at 383nm was blue shifted and increased in intensity in the M13_EGFR_(TNP), due to the formation of the amidic bond between the TM derivative and the amine groups of the M13_EGFR_ phage (See Figures S6 and S7, Supporting Information). M13_EGFR_(TNP) preserved also the typical fluorescence of the oligothiophene monomer (Figure 3C) that is strongly red‐shifted (from 603 nm to 640 nm) upon conjugation, owing to the planarization effect of the oligothiophene molecule induced by the phage scaffold. Notably, this red‐shift is extremely attractive for imaging applications thanks to an improved tissue penetration and reduced background interference.

The ability of M13_EGFR_(TNP) to act as a photosensitizer in response to visible light exposure, was evaluated using the Amplex Red and ABMDMA assays.

Photosensitizers can absorb light at a specific wavelength and be promoted from their ground state (S_0_) to the first singlet excited state (S_1_). T_1_ excited state can be populated via intersystem crossing from S_1_ to T_1_. The T_1_ excited state is generally characterized by a long lifetime and can interact with molecular oxygen through two alternative mechanisms: i) type I mechanism, based on electron transfer, that generates different radical oxygen species and hydrogen peroxide; ii) type II mechanism, based on energy transfer, where singlet oxygen is produced.

The Amplex red assay quantifies the production of peroxides upon irradiation (type I mechanism), by measuring the appearance of a fluorescence signal, due to the resorufin molecule formed by the reaction of the nonfluorescent Amplex Red with peroxides, catalyzed by HRP.

The ABMDMA assay determines the singlet oxygen generated during visible light irradiation (type II mechanism) following the decrease of ABMDMA absorbance upon irradiation: the singlet oxygen reacts with ABMDMA to give a non‐absorbing endoperoxide.

**Figure 2:**
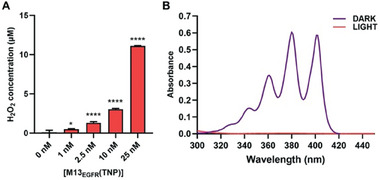
Photo‐dependent ROS generation of M13_EGFR_(TNP). A) Peroxide generation was estimated by measuring the fluorescence of resurfin, while B) singlet oxygen production was evaluated by measuring the decrease of the absorbance of the ABMDMA molecule. Statistical significance was calculated by one‐way ANOVA in comparison to the control (0 nm) ^*^ = *p*<0.05, ^****^ = *p*<0.0001.

**Figure** **2** shows that, upon irradiation, M13_EGFR_(TNP), is able to generate peroxides (type I mechanism) in a concentration‐dependent manner. At the same time M13_EGFR_(TNP) efficiently produces ^1^O_2_ (type II mechanism). Due to the singlet oxygen produced during the irradiation, M13_EGFR_(TNP) causes a complete degradation of ABMDMA, even at the lowest concentration tested of 1 nm. These results prove that M13_EGFR_(TNP) is characterized by a high photoactivity, following both type I and type II mechanisms in ROS production.

### 2.3 Determination of the Tropism of M13_EGFR_(TNP)

M13 phages with targeted tropism against EGFR (M13_EGFR_) can be developed through specific display on the minor coat protein pIII of SYPIPDT peptides.^[^ ^80^, ^81^ ^]^ EGFR was selected as a target because its overexpression is observed in many cancers and EGFR‐targeted therapies have great anticancer potential.^[^ ^83^ ^]^

The maintaining of the targeting activity of M13_EGFR_ against EGFR was assessed for M13_EGFR_(TNP) using confocal microscopy and flow cytometry assays, in order to rule out any impairment of vector targeting ability following the use of the viral capsid as templating agent.

To test the EFGR‐recognition ability of the M13_EGFR_(TNP), the human epidermoid carcinoma cell line A431 was used, because it expresses high levels of EGFR. The HSA bioconjugate (HSA‐TM), missing any ability to target EGFR, was also investigated as negative control.

**Figure 3:**
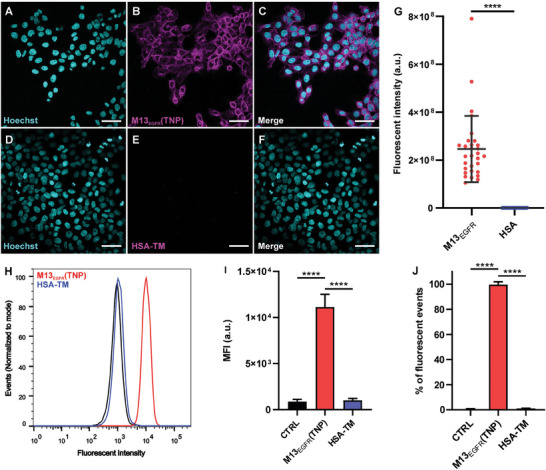
Targeting of the M13_EGFR_(TNP) and HSA‐TM bioconjugates on A431 cell line. Confocal microscopy images of cells after incubation for 45 min with M13_EGFR_(TNP) (A–C) or HSA‐TM bioconjugates (D–F) at equivalent carrier concentration (1 nm). A) and D) nuclei colored in cyan, B) and E) oligothiophene fluorescence in magenta, C) and F) merged images. Scale bar = 50 µm. G) Semi‐quantitative analysis of oligothiophene fluorescence intensity detected in confocal images. Flow cytometry results expressed as H) histogram of fluorescence peaks, I) mean fluorescence intensity (MFI), J) percentage of fluorescent events. Statistical significance was calculated by *t*‐test for the analysis performed on confocal images and by one‐way ANOVA multiple comparison for flow cytometry data, ^****^ *p*<0.0001.

A431 cells show a very efficient internalization of the M13_EGFR_(TNP) within 45 min, while poor internalization was observed for the HSA‐TM bioconjugate (**Figure** **3**A–F). This difference appeared evident by analysis performed on microscopy images. Indeed, fluorescence in the oligothiophene channel increased by three orders of magnitude on A431 incubated with M13_EGFR_(TNP) compared to cells incubated with equimolar concentrations of HSA‐TM bioconjugates (Figure 3G). Comparable effects were obtained by flow cytometry, where i) a clear shift in the fluorescence mode (Figure 3H), ii) an increase in the mean fluorescence intensity (Figure 3I) and iii) a raise in the percentage of fluorescent cells (Figure 3J) were detected only in A431 cells treated with M13_EGFR_(TNP).

**Figure 4:**
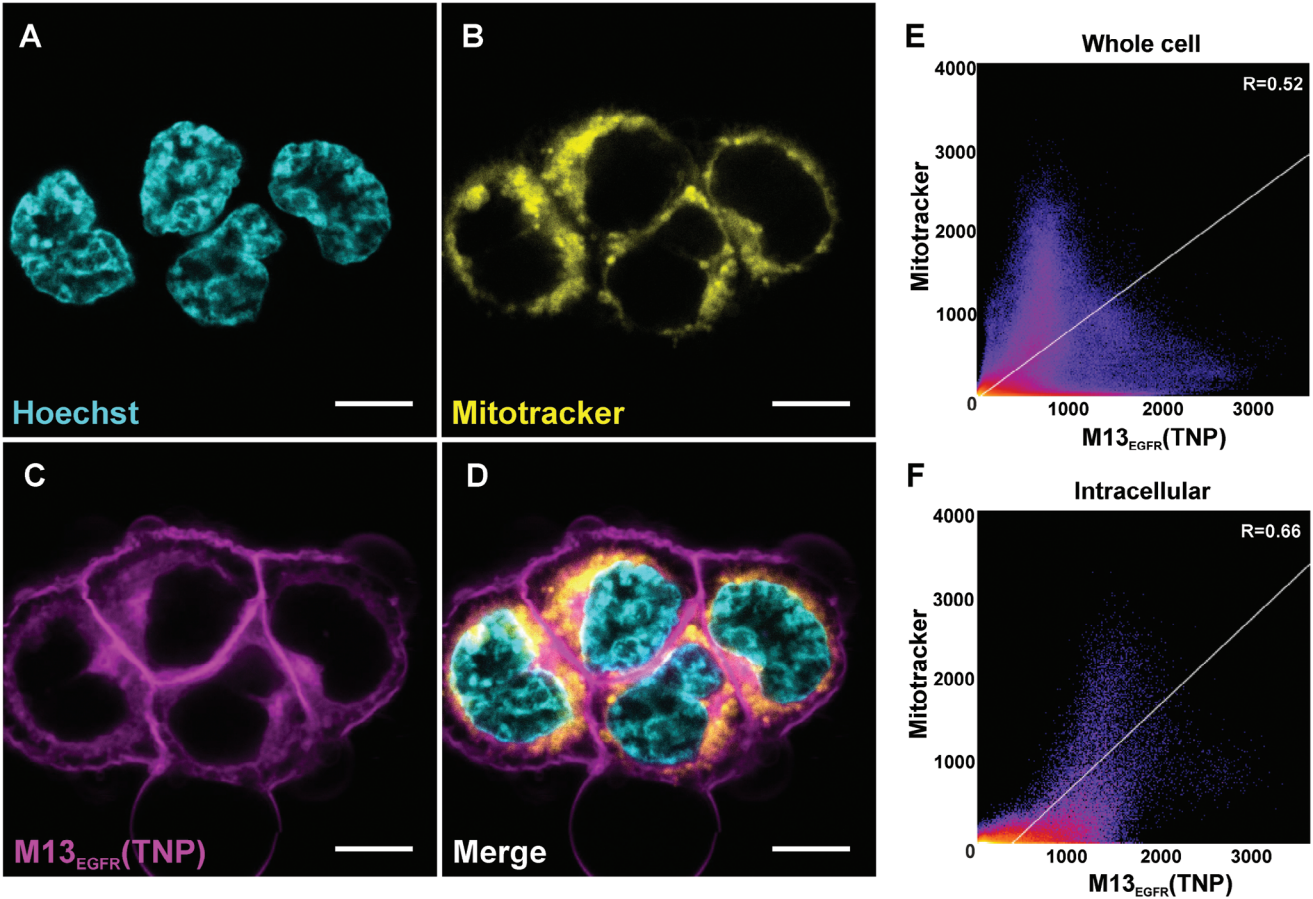
Cellular localization of M13_EGFR_(TNP) on A431 cell line. Confocal microscopy images of cells stained with Mitotracker and incubated with M13_EGFR_(TNP) (A–D). A) nuclei counterstained by Hoechst are shown in cyan, B) Mitotracker staining, yellow, C) oligothiophene fluorescence, magenta, D) merged images from A, B, and C. Scale bar, 10 µm. (E, F) Scatter plots representing colocalization of Mitotracker and M13_EGFR_(TNP) on A431 cells. Colocalization was determined in the whole cell area (E) or intracellularly (F) by excluding the signals on the cell membrane. In E‐F panels, white line is the tendency line and R show Pearson's R value calculated with Fiji Coloc2.

The cellular localization of the delivered M13_EGFR_(TNP) was investigated (**Figure** **4**) by confocal microscopy. M13_EGFR_(TNP) tends to accumulate on cell membrane and, intracellularly, in the perinuclear region. In particular, intracellular M13_EGFR_(TNP) overlayed with the Mitotracker fluorescence signal. A colocalization analysis using the FIJI Coloc 2 plugin showed a correlation, with a Pearson's R value equal to 0.66 for M13_EGFR_(TNP), as already observed for the nude M13_EGFR_ phage. This localization is particularly interesting because mitochondria are organelles that are highly susceptible to photodamage, and their tageting may in principle enhance the effects of phototherapies.^[^ ^84^ ^]^

These results demonstrated that, i) the phage‐templated synthesis of TNP preserves the recognition ability of the phage, ii) the M13_EGFR_(TNP) is efficiently internalized by EGFR‐overexpressing cells, iii) the presence of M13_EGFR_(TNP) at the cell surface argues in favour of a receptor‐mediated internalization of M13_EGFR_(TNP), iv) M13_EGFR_(TNP) co‐localizes with mitochondria offering the possibility to amplify the damages induced by the ROS production upon irradiation.

### 2.4 Photoactivity of M13_EGFR_(TNP) in 2D Cell Cultures

The performances of M13_EGFR_(TNP) in PDT were tested on the EGFR‐overexpressing A431 cell line. HSA‐TM bioconjugates were also tested as a non‐targeted control carrier.

**Figure 5:**
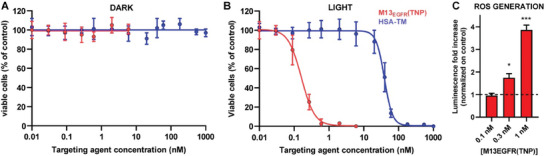
Photo‐dependent cytotoxicity on cancer cells treated with M13_EGFR_(TNP) or HSA‐TM bioconjugates. A431 cells incubated for 45 min with M13_EGFR_(TNP) or HSA‐TM bioconjugates, were A) kept in dark condition or B) irradiated for 10 min with white light, and cell viability was evaluated 24 h after the treatment. Data are shown as mean ± SD of 3 independent experiments and results are expressed as percentage of control (untreated – dark). C) Photo‐induced intracellular ROS generation in cancer cells treated with increasing concentrations of M13_EGFR_(TNP). Results are expressed as luminescence fold increase normalized on control (untreated). Statistical analysis was performed using one‐way ANOVA in comparison to the control (0 nm), ^*^ *p*<0.05, ^***^ *p*<0.001.

M13_EGFR_(TNP) is biocompatible and does not exhibit any “dark toxicity,” as there was no discernible decrease in viability for A431 cells maintained in dark conditions (**Figure** **5**A), when incubated with increasing concentrations of M13_EGFR_(TNP). In contrast, upon irradiation with visible LED light, even at an ultralow light dose (24.0 mW cm^−2^), M13_EGFR_(TNP) displayed a dose‐dependent cytotoxic effect on A431 cells, demonstrating the efficiency of M13_EGFR_(TNP) in PDT (Figure 5B). Complete killing of the cancer cells was observed at 1.0 nm concentration of M13_EGFR_(TNP) upon irradiation, while the concentration of HSA‐TM, had to be increased up to 180 nm to obtain the same effect. This remarkable difference was reflected and highlighted also by the calculation of the IC_50_, which is 0.16 nm for M13_EGFR_(TNP) and 38.6 nm for the HSA‐TM bioconjugate. The primary mechanism of PDT‐induced cancer cell death is generation of ROS‐mediated damages. To test the cytotoxic oxidative stress induced by M13_EGFR_(TNP) in A431 cells, upon illumination, we used ROS‐Glo™ as a reporter to monitor the intracellular formation of reactive oxygen species (ROS). Significantly, ROS generation increased only upon PDT treatment; in particular, we recorded a 1.5‐ and 3.5‐fold increase in A431 cells treated with 0.3 and 1 nm of M13_EGFR_(TNP), compared to untreated cells (Figure 5C). Generation of ROS (Figure 5C) coincides with the appearance of a significant cytotoxic effect on the cell line (Figure 5B), suggesting that the intracellular generation of ROS, mediated by the irradiation of M13_EGFR_(TNP), promotes the observed cell death.

**Figure 6:**
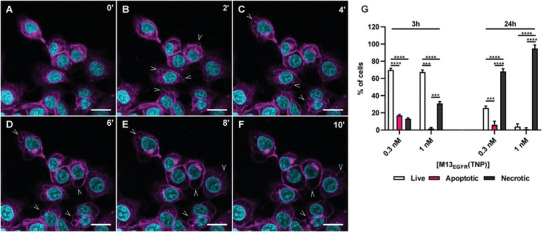
Cell damage and mechanism of cell death upon bioconjugates irradiation. Real‐time monitoring of the photodamage induced by M13_EGFR_(TNP) on A431 cells over time. Images were acquired every 2 min from A) t = 0 min to F) t = 10 min. Panels A‐F are merged images, nuclei are stained with Hoechst and colored in cyan while M13_EGFR_(TNP) is colored in magenta. Scale bar = 20 µm. G) Evaluation of cell death mechanism. Percentage of living cells (white), cells undergoing apoptosis (purple), and necrotic cells (dark grey), 3 and 24 h after PDT treatment with M13_EGFR_(TNP). Results are expressed as mean± SD of 3 independent experiments. Statistical significance was calculated by one‐way parametric ANOVA followed by Dunnet's multiple comparison test; ^***^ *p*<0.001; ^****^ *p*<0.0001; *n* = 3.

The photodamage mediated by M13_EGFR_(TNP) on cancer cells was further investigated by time lapse confocal microscopy (**Figure** **6**A–F). Images of A431 cells were acquired every 2 min, irradiating with white light between each acquisition. Over time, an evident membrane blebbing process was observed, with the formation of small membrane extrusions just after 2 min of irradiation, which increased in size in course of the experiment. The membrane blebbing phenomenon, previously observed also for the HSA‐TM bioconjugate on HeLA cells,^[^ ^79^, ^85^ ^]^ is a structural modification induced by oxidative stress that can be associated with different forms of cell death.

Flow cytometry analysis (Figure 6G) of 7‐aminoactinomycin (7‐AAD) and annexin V was performed to investigate the mechanism of cell death induced by photoactivation of M13_EGFR_(TNP).

3 h after the irradiation 16.95% of cells were apoptotic, 13% necrotic and 69.7% alive, while at 24 h necrosis predominated, accompanied by a remarkable decrease in cell viability.

Altogether these results demonstrated the theranostic potential of the M13_EGFR_(TNP) platform, able to detect EGFR‐positive cancer cells and simultaneously induce their controlled ablation.

### 2.5 Photoactivity of M13_EGFR_(TNP) in 3D Spheroids

The culture of cancerous cells in standard monolayer conditions poorly mirrors the physiology (i.e., the drug sensitivity) of tissues or organs. Accordingly, the interest in 3D cellular models has raised in recent years, highlighting diverse responses to PDT between 2D and 3D cell models.^[^ ^85^ ^]^

**Figure 7:**
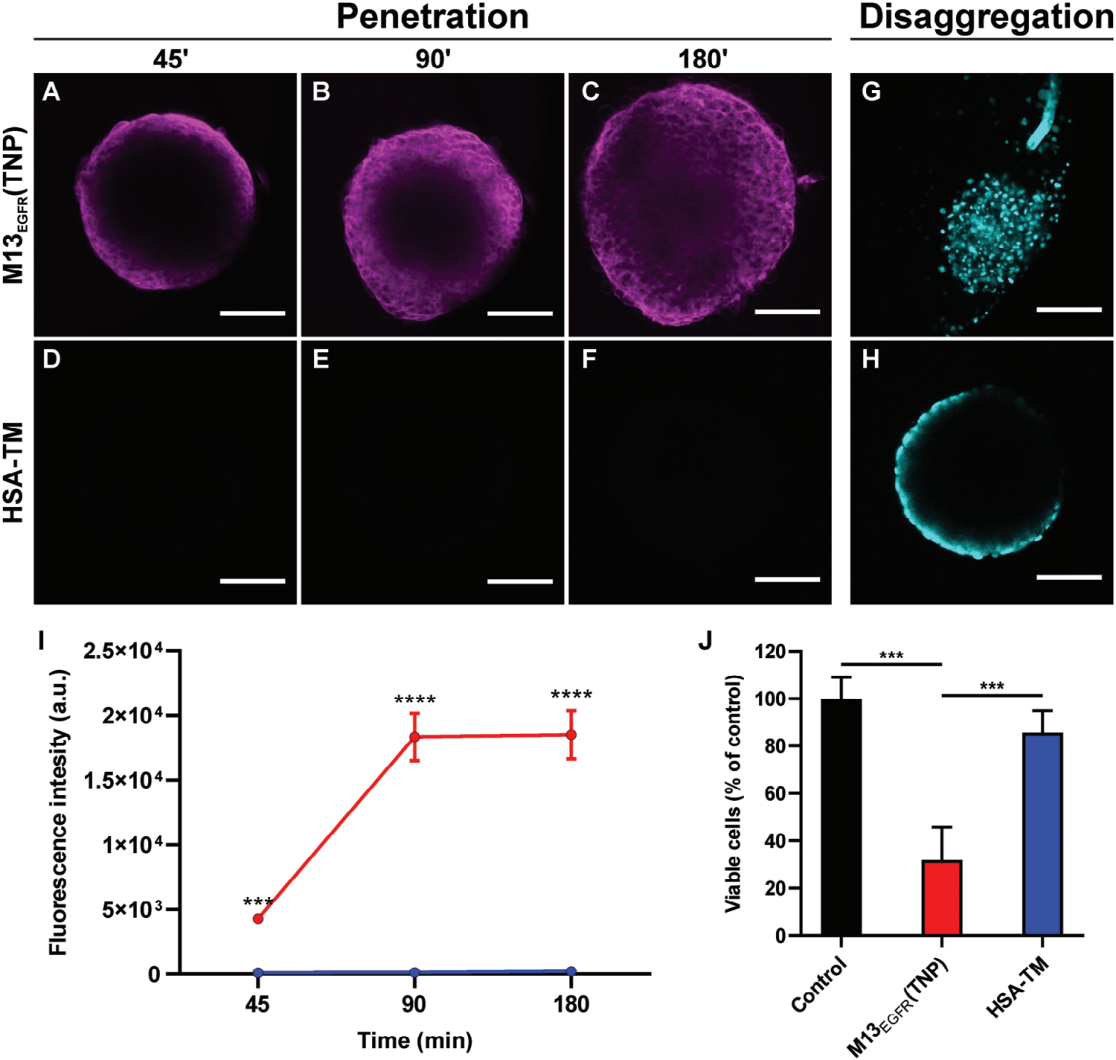
Targeting and phototoxicity of M13_EGFR_(TNP) and HSA‐TM bioconjugates on 3D spheroids. Penetration into spheroid of A–C) M13_EGFR_(TNP) and D–F) HSA‐TM bioconjugates at a concentration of 3 nm, over time. Acquisitions were performed every 45 min for 180 min and laser settings were maintained fixed among different samples. Fluorescence of the oligothiophene derivatives is shown in magenta. Scale bar = 100 µm. I) Quantification of fluorescence intensity detected in panel (A–F). Spheroid integrity after PDT was assessed 24 h after the treatment with G) M13_EGFR_(TNP) or H) HSA‐TM bioconjugates, by acquiring confocal images of Hoechst (cyan) labeled spheroid. J) Spheroid viability evaluated through CellTiter‐Glo 3D Cell Viability Assay 24h after PDT treatment. Statistical analysis was performed by one‐way ANOVA multiple comparison. ^***^ *p*<0.001, ^****^ *p*<0.0001.

The capability of M13_EGFR_(TNP) and HSA‐TM bioconjugates to penetrate tumoral 3D structures was evaluated on A431 spheroids through confocal microscopy (**Figure** **7**). M13_EGFR_(TNP) penetrance into spheroid increases over time, reaching the compact necrotic core after 3 h from the incubation (Figure 7). Conversely, HSA‐TM bioconjugates poorly permeated the A431 spheroids, as shown by the lower fluorescence signals in the oligothiophene channel, compared to spheroid treated with M13_EGFR_(TNP).

These results reveal that M13_EGFR_(TNP) is able to deeply penetrate into the spheroid core, where other therapeutic molecules or platforms (such as antibodies, VLPs, etc.) typically fail. This is a feature that is ideal for PDT applications.^[^ ^85^ ^]^

The same experiments were carried out with i) a 1D‐TNP, missing of the EGFR recognition moiety, using the wild‐type M13 phage as a template, that is, M13(TNP) and ii) 3D‐TNPs (Figure S8, Supporting Information). M13(TNP) is able to penetrate the outer cell layers of the spheroid, but the penetration depth is limited, while 3D‐TNPs, not surprisingly, are totally incapable to penetrate the spheroid.

The results showed that the ability of the M13_EGFR_(TNP) to deeply penetrate spheroids is due the innate capability of filamentous phages to penetrate tissues and barriers,^[^ ^46^, ^50^, ^51^ ^]^ due to their aspect ratio, in combination to the EGFR‐mediated recognition processes of the refactored M13_EGFR_ phage.^[^ ^46^, ^50^, ^51^ ^]^

The effect of phage‐mediated PDT was evaluated by looking for possible changes in the spheroid morphology, as well as by determining the cell viability after irradiation. In terms of spheroid integrity, a clear loss of the 3D structure with the presence of numerous single cells detached from the spheroid was observed after photoactivation of M13_EGFR_(TNP). The only supracellular structure still present 24 h after the treatment was the inner compartment of the spheroid, usually described as the necrotic core. In terms of cell viability, the percentage of viable cells decreased by 70% in comparison to the control, after PDT with M13_EGFR_(TNP) at the final concentration of 3 nm, while HSA‐TM bioconjugates did not produce any significant effect at the same concentration.

### 2.6 In vivo Nanosafety of M13_EGFR_(TNP)

Following the in vitro assessment of the biosafety of M13_EGFR_(TNP) in dark conditions, in vivo assays were performed with *Hydra vulgaris*, a model organism successfully employed to test toxicity and bioactivity of both inorganic and organic compounds. *Hydra* polyps habit clear lake waters and are very sensitive to toxicants present in the surrounding medium, making it an extraordinary model for environmental pollution studies. In case of exposure to heavy metals, for instance, animal behaviors (contractions, swelling of extended body regions) and tissue disintegration can be monitored and quantified according to well established methods.^[^ ^86^, ^87^, ^88^ ^]^

**Figure 8:**
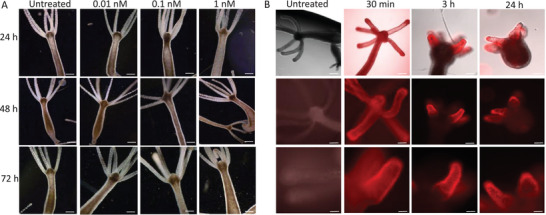
Biosafety and biodistribution of M13_EGFR_(TNP) in *Hydra vulgaris*. A) Toxicological assessment of engineered phages in dark condition. Polyps were continuously incubated with M13_EGFR_(TNP) at the indicated concentrations (related to the bacteriophage) and up to 72 h. Data show biosafety in every condition at every time point. Each image shows a representative polyp of a given condition. All polyps analyzed (n = 10) exhibited the same phenotype. Scale bars: 500 µm. B) In vivo biodistribution of M13_EGFR_(TNP) in *Hydra* by fluorescence imaging. Untreated polyps show the absence of tissue autoflorescence in the spectral region selected by the fluorescence microscopy filter set (BP365/12‐FT395‐LP397). Polyps treated with 0.1 nm M13_EGFR_(TNP) were observed after 30 min, 3 h or 24 h treatment. Each column shows for each incubation time the brightfield‐fluorescence merged images (upper row) and the fluorescence images at two magnifications of a representative polyp. 10 polyps per condition were analyzed, showing the same strong red fluorescence labeling preferentially the tentacles. Scale bars, 500 µm (upper and middle rows); 100 µm, lower row.

Toxicity assays were carried out by treating *Hydra* with M13_EGFR_(TNP) at three different concentrations (0.01, 0.1 and 1.0 nm) and continuously monitored for the appearance of tissue damages, detectable by tissue morphological alteration and integrity of the cells in the body column or in the tentacles. As shown in **Figure** **8**A, treated polyps did not present signs of damage, at any time of continuous incubation, suggesting the biosafety of the engineered compound in absence of photostimulation.

Next, we evaluated the in vivo biodistribution of M13_EGFR_(TNP) in *Hydra vulgaris*. The polyp body transparency allows to detect in living specimens the localization of fluorescent proteins or to follow the dynamic of the internalization of fluorescent nanosized compounds. The 0.1 nm dose was selected to guarantee a good staining. After 30 min of incubation the labeling was uniform on the tentacles and on the body column, indicating an initial absorption of the phage on the cell surface (Figure 8B). This uniform labeling has been observed by treating *Hydra* for the same incubation period with quantum rods and it was shown to be enhanced in case of positively charged nanoparticles.^75^ After 3 h of incubation the red fluorescence of the oligothiophenes was detected very clearly on the tentacles and less on the body column region, indicating a preferential uptake and accumulation of M13_EGFR_(TNP) in these regions. Remarkably, after 24 h, a granular staining was observed in the tentacles, possibly indicating the accumulation of M13_EGFR_(TNP) into storage vacuoles, mirroring the internalization dynamics observed with other nanoparticles such as quantum rods and gold nanoparticles.^[^ ^89^, ^90^ ^]^

### 2.7 In Vivo Photodynamic Treatment by using M13_EGFR_(TNP)

**Figure 9:**
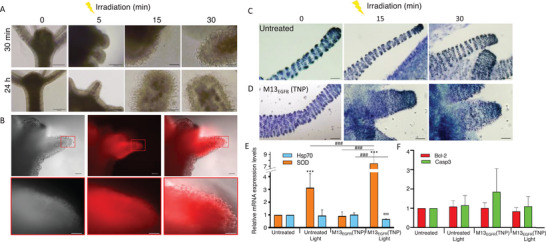
In vivo photodynamic treatment with M13_EGFR_(TNP). A) Polyps were treated with 0.1 nm of M13_EGFR_(TNP) and irradiated (light power density of 0.04 mW cm^−2^) at different durations. The images show intact head and body tissue of treated polyp before irradiation (left column) and after 5, 15, and 30 min of irradiation. Cell blebbing and damages of tentacle cells increase progressively as the irradiation time increases. 10 polyps/conditions were analyzed, showing the same damages on all tentacles. Scale bars, 200 µm (0 min and 5 min irradiation), 50 µm (15 min and 30 min irradiation) B) In vivo fluorescence imaging of a *Hydra* during photodynamic treatment. Brightfield (left column), fluorescence (middle), and merged (right column) images of a representative *Hydra* polyp treated 30 min with the M13_EGFR_(TNP) and irradiated for 10 min. The lower panel shows details of the region red framed in the upper panel. Clear cell lysis occurs on the tentacles, co‐localizing with the M13_EGFR_(TNP) fluorescence. Scale bars, 100 µm. C) Toluidine‐blue staining of tentacles showing the nematocytes organized in battery cells. D) The organization and the number of nematocytes is depleted in animals treated with M13_EGFR_(TNP) and progressively increase with the irradiation time. Scale bars, 200 µm. E,F) Transcriptional analysis of stress and apoptotic responsive genes in response to photodynamic treatment. Animals were treated 30 min with 0.1 nm phage, washed, irradiated 10 min, and allowed to recover 4 h before processing for RNA extraction and qRT‐PCR analysis using specific primers (see Table S1, Supporting Information). The *Hydra Elongation factor 1‐alpha* (*Ef‐1α*) was used as reference gene. Transcription levels of E) *HySOD* and *Hsp70*, and F) *HyCasp‐3* and *HyBcl2* genes. Data represent the mean ±SD of three technical repeats from two biological replicates (n = 15). An unpaired *T*‐test was used for statistical comparisons. ^*^ *P*<0.05, ^**^ *P*<0.01, ^***^ *P*<0.0001.

To investigate the capability of M13_EGFR_(TNP) to induce cell death in vivo, *Hydra* polyps were treated with the same phage dose used for biodistribution studies (0.1 nm), and two periods of treatment were tested, that is, 30 min and 24 h, to take into account for possible differences in the photostimulation efficacy due to the localization of the M13_EGFR_(TNP) either on the cell surface (30 min treatment) or the penetration inside the ectodermal cells (24 h treatment). After treatment and extensive washing to eliminate unbound M13_EGFR_(TNP), polyps were individually irradiated to activate M13_EGFR_(TNP). As shown in the **Figure** **9**A, clear signs of cell blebbing and lysis were observed all around the tentacles already after 5 min of irradiation, and the entity of this damage increased over time. These results closely resemble the in vitro experiments showed in the 2.2 section. Remarkably, in each polyp, all tentacles appeared similarly damaged (6/6), due to the irradiation spot size illuminating the whole animal, and the efficiency of the treatment was highest (10/10 treated polyp). The possibility that the irradiation itself may cause tissue damages was tested by irradiating for the same periods living polyps in absence of any phage treatment. Figure S10 (Supporting Information) show that the tissue morphology and integrity was not affected by irradiation, confirming the phage specific phototoxicity.

In vivo fluorescence imaging during photodynamic treatments confirms the tentacle specific effect of M13_EGFR_(TNP) in the polyps, the cell lysis occurring in red stained regions (Figure 9B).

The dependency of the observed cell lysis from the M13_EGFR_(TNP) was demonstrated by treating *Hydra* with the same phage, but conjugated with a fluorophore, not acting as photosensitizer, that is, M13_EGFR_‐CF488A. Following toxicological tests to ensure biosafety of the phage bioconjugate (Figure S11, Supporting Information), living polyps were treated with the same phage dose employed for M13_EGFR_(TNP) and irradiated for the same periods. Figure S12 (Supporting Information) shows the intact *Hydra* epithelium observed at all conditions, indicating the absence of effects from the generic fluorophore, and confirming the specific cell ablation observed, upon irradiation, with M13_EGFR_(TNP).

In *Hydra* species VEGF and FGF homologues and their receptors VGFR2 and FGFR‐1 have been isolated and characterized.^[^ ^91^, ^92^ ^]^ In addition, several other components of the receptor tyrosine kinase family and relative signaling pathway components have been identified, showing considerable structural similarities with their human homologues,^[^ ^93^, ^94^ ^]^ that is the presence of three extracellular immunoglobulin domains (D1‐D3), a transmembrane domain and an intracellular tyrosine kinase domain. These features are present for instance in the HyFGFR‐1 protein structure, which expression has been found in the tentacles and body column cells. The selective tropism of the M13_EGFR_(TNP) for tentacle cells might be explained with binding of a specific EGFR‐like protein present on the tentacle cells, and this hypothesis is supported by the aberrant morphology showed by tentacle cells following photostimulation. By using Toluidine blue to stain a specific cell type peculiar of the cnidaria phylum, the nematocyst, the number and the distribution of the nematocysts following phage treatment and irradiation appear strongly affected (Figure 8D), compared to the highly ordered distribution of nematocytes on untreated animals (Figure 8C). This aberrant morphology clearly indicates the tentacle cells as targets of M13_EGFR_(TNP) photoactivation.

Gene expression analysis through real time quantitative Reverse Transcription Polymerase Chain Reaction (qRT‐PCR) was performed to identify the molecular pathways (oxidative stress and apoptosis) possibly modulated by M13_EGFR_(TNP)‐mediated photodynamic treatment. *Superoxide Dismutase (SOD)* and *Heat Shock Protein‐70 (Hsp70)* genes were selected due to their well characterized expression profile in *Hydra* in response to oxidative and thermal stresses mediated by gold and iron oxide nanoparticles.^[^ ^95^, ^96^ ^]^ Following the M13_EGFR_(TNP)‐mediated photodynamic treatment, *SOD* expression was found slightly upregulated as effect of the light exposure alone, which is reasonable, since it is known an increased ROS production after irradiation at the wavelengths employed for the photostimulation (photobiomodulation). In presence of the M13_EGFR_(TNP) photoactivation SOD was strongly up‐regulated, due to increased ROS production during the PDT treatment.

A slight down‐regulation was observed for *Hsp70* (Figure 9E). *Caspase3* (*Casp3*) and *Bcl‐2‐like 4* gene expression were also evaluated as apoptosis markers. *Casp‐3*, a pro‐apoptotic gene activated downstream the molecular cascade, and *Bcl‐2* an anti‐apoptotic genes, have been both characterized in *Hydra* in homeostatic conditions and in response to heavy metal exposure.^[^ ^87^, ^97^, ^98^ ^]^ Following the photodynamic treatment, no significant modulations of their expression were observed after a reasonable period of time (4 h) necessary to observe transcriptional changes (Figure 9F). The absence of modulation within the observed temporal window suggests that the morphological damages observed during the 30 min irradiation are due to necrosis or later stages of a regulated cell death mechanism, as already observed in in vitro experiments.

## 3 Conclusion

Nanobiotechnology integrates the peculiar physico‐chemical properties of nanomaterials with biosystems.^[^ ^99^, ^100^ ^]^ Engineered living materials (ELM)^[^ ^101^ ^]^ focuses on novel hybrid materials derived from combination of artificial and biological components, presenting the hallmarks of material from nature, from biocompatibility to targeting, adaptation, longevity and sustainability. Here we leveraged on the unique properties of bacteriophages (protein‐based structure, possibility to be genetically engineered, tropism for specific cell types) to biofabricate a novel hybrid nanostructured material, biocompatible, stable, highly photoactive and receptor‐specific. We transformed a natural entity, that is, a refactored filamentous M13 phage, reprogrammed to target EGFR (M13_EGFR_), into a nanodevice for receptor‐targeted PDT and imaging. The synthetic process generated a monodispersed, uniformly sized 1D‐TNP, namely M13_EGFR_(TNP), that fully maintains the morphological characteristics of the template phage. M13_EGFR_(TNP) is characterized by red fluorescence, a property that is extremely attractive for imaging applications. Remarkably, M13_EGFR_(TNP) was shown highly efficient to generate peroxides and singlet oxygen species upon irradiation. In vitro experiments showed that i) the phage‐templated synthesis of TNP preserves the recognition ability of the refactored phage, ii) an efficient receptor‐mediated internalization of the M13_EGFR_(TNP) was observed in cells overexpressing EGFR. iii) M13_EGFR_(TNP) was biocompatible and did not exhibit any “dark toxicity.” In contrast, upon irradiation with visible LED light, even at an ultralow light dose, M13_EGFR_(TNP) demonstrated excellent performances in PDT, with a killing activity at picomolar concentrations (IC_50_ = 160 pm). To the best of our knowledge, this is one of the lowest concentrations ever observed for PDT treatment. The M13_EGFR_(TNP)‐mediated photodamage on cancer cells was investigated in real time. An evident membrane blebbing process, induced by oxidative stress and associated with apoptosis was observed over time. Going one step further in the complexity of the biological system, we tested the engineered bacteriophage in 3D cellular cultures, i.e spheroids from A431 cells. M13_EGFR_(TNP) was able to deeply penetrate the spheroid core and break up, upon irradiation, its supracellular structure. Finally, switching from in vitro biosystems to in vivo whole animal system, M13_EGFR_(TNP) was tested in a tissue‐like organism, *Hydra vulgaris*, recognized as unique model to test biosafety and bioactivity of a multitude of nanostructured devices. We showed that in absence of photostimulation M13_EGFR_(TNP) did not induce any toxicity in *Hydra* tissue. The presence of EGFR drives the vector in the tentacles, showed by in silico analysis to express putative *Hydra* EGFR homologues. Following the incubation of whole polyps with M13_EGFR_(TNP), irradiation induced a strong disorganization of tentacle specific cells, nematocytes, and the appearance of cell blebbing all over the tentacle length. Remarkably, from gene expression analysis, a strong upregulation of the antioxidant enzyme SOD was observed specifically in treated and irradiated animals, suggesting a clear response of the animal to reestablish redox equilibrium through the activation of the detoxifying genes. Interestingly, the genes involved in the programmed cell death, like *Bcl2* and *Casp‐3*, were found not significantly modulated, indicating the induction of a fast necrosis process rather than a slower apoptotic pathway by the M13_EGFR_(TNP) photostimulation.

In conclusion the developed methodology provides an extremely efficient phototheranostic platform able to detect, image and ablate cells/tissues overexpressing EGFR.

The nanoarchitectonics approach employed for M13 functionalization and targeting may have a more broadly application: i) M13 phage may be genetically reprogrammed to target different cell types, from cancer cells to bacterial cells, and thus exploited for anticancer as well for antimicrobial strategies;^[^ ^102^ ^]^ ii) the morphology of the desired TNP may be finely controlled by selecting the viral template, and this in turn may drive the synthesis of TNP with well‐defined shapes and desired size; iii) the chemo‐physical properties of the TNP may be easily tuned by molecular engineering of the oligothiophene unit used to synthesize the TNP; iv) different semiconducting nanoparticles with tailored properties, can be synthesized, replacing the oligothiophene derivatives with diverse molecular scaffolds, opening up new scenarios for light controlled therapies/imaging procedures.

## 4 Experimental Section

### Retargeting of M13 Phage Against EGFR

The recombinant M13, expressing an EGFR binding peptide (SYPIPDT) in fusion with the pIII phage protein (M13_EGFR_), was genetically engineered by using phagemid pPK15, recently described by Ulfo and colleagues.^[^ ^80^ ^]^ Phage production was performed via infection of *E. coli* TG1 cells carrying pIII modified expressing plasmid (pPK15) with Hyperphage helper (ProGen), and purification of recombinant phages was achieved through multiple precipitations with PEG and isoelectric point.^[^ ^103^ ^]^ In particular, bacteria were grown in LB liquid supplemented with Kanamycin (25 mg L^−1^), Ampicillin (100 mg L^−1^), and 0.4 mm IPTG for 24 h. Next, the bacterial culture was centrifuged for 20 min at 12 000 g and the obtained supernatant was supplemented with PEG 8000 (4% w/v) and NaCl (3% w/v). After 1 h of incubation on ice, the solution was centrifuged for 20 min at 12 000g and the pellet gently resuspended in PBS 1x. Phages present in the solution were precipitated by lowering pH to 4.2, corresponding to M13 isoelectric point (IEP), and centrifuging at 12 000 g for 10 min. M13_EGFR_ was finally resuspended in PBS 1x and the phage concentration was deduced by measuring the absorbance at 269 nm in a UV–vis spectrophotometer, using an extinction coefficient of ε = 3.84 cm^2^ mg^−1^.

### Synthesis and Purification of the Phage‐Templated Thiophene Nanoparticles M13_EGFR_(TNP)

Oligothiophene derivatives were dissolved in DMF to obtain a concentration of 5 mm. 50 µL of this solution was slowly added dropwise to 1 mL of M13_EFGR_ 40 nm in sodium carbonate buffer 100 mm pH 9 under vigorous stirring.

The reaction was incubated for 3h at 25 °C under constant shaking at 700 rpm (ThermoMixer HC, S8012‐0000; STARLAB, Hamburg, Germany) in the dark, and then it was centrifuged at 14000g for 10 min to remove the insoluble excess of nonconjugated oligothiophene derivatives. The sample was then extensively dialyzed against PBS 1x in cellulose membrane dialysis tubes with a 14 kDa cut‐off to remove the water‐soluble byproducts generated during the coupling procedure. Finally, the sample was again centrifuged at 14000g for 10 min to ensure the total removal of the insoluble oligothiophene derivative. Synthesis and characterization of HSA‐TM were previously reported.^[^ ^79^ ^]^

### Spectroscopic Characterization of M13_EGFR_(TNP)

For the spectroscopic characterization of M13_EGFR_(TNP), absorption spectra were recorded using a Cary60 UV–vis spectrophotometer (Agilent). The emission spectra were acquired with an Edinburgh FLS920 spectrometer, equipped with a photomultiplier Hamamatsu R928P.

### Determination of the ROS Generation Ability of M13_EGFR_(TNP) upon Irradiation

Amplex Red Assay. Nonfluorescent Amplex Red, reacts with peroxides to form fluorescent resorufin, catalyzed by HRP. The difference between the fluorescence of the resorufin produced by the irradiated samples and the non‐irradiated references – that are identical solutions stored in the dark – is used to evaluate the concentration of the peroxides that are formed.

90 µL of solutions of 3D‐TNP and M13_EGFR_(TNP) at different concentrations (0, 1, 2.5, 10, and 25 nm) in phosphate buffer (PB) 50 mm pH 7.4, were irradiated for 30 min with visible light (Valex cold white LED, irradiance on the multiwell plate surface = 24.0 mW cm^−2^). For every sample, three technical replicates were carried out. A working solution (WS) was obtained by diluting 10 µL of Amplex Red (AR) stock solution 50 mm prepared in DMSO to 1 mL of 50 mm PB 50 mM (pH 7.4), obtaining a concentration of AR of 500 µm. 10 µL of HRP 0.4 mg ml^−1^ in PBS were also added to the WS. After irradiation, 10 µL of the WS was added in each well and left, in the dark, to incubate for 30 min at room temperature. Following incubation, using a PerkinElmer EnSpire® Multimode Plate Reader, the fluorescence intensity of the solutions was measured at 590 nm (*λ*_ex_  = 560 nm). The fluorescence signal was converted to the concentration of peroxides generated upon irradiation, using a calibration curve created using standard solutions of H_2_O_2_.

ABMDMA Assay. 9,10‐Anthracenediyl‐bis(methylene) dimalonic acid (ABMDMA) reacts with singlet oxygen to give an endoperoxide. The bleaching of ABMDMA is used to quantify the ^1^O_2_ produced upon irradiation. Solutions at different concentrations of 3D‐TNP and M13_EGFR_(TNP) (0, 1, 2.5, 10 and 25 nm) were prepared in deuterated PBS 1x. A 96‐multiwell plate was loaded with 96 µL of each solution and mixed with 4 µL of ABMDMA stock solution (5 mM in DMSO). Successively, the plate was exposed for 30 min to a visible light lamp (Valex cold white LED, irradiance on the multiwell plate = 24.0 mW cm^−2^). At the end of the irradiation the UV–vis spectrum of the solutions was measured at 380 nm to quantify the remaining ABMDMA using an EnSpire® Multimode Plate Reader. For every sample, three technical replicates were carried out.

### Cryo‐TEM of M13_EGFR_(TNP)

Aliquots of 3 µL of M13_EGFR_(TNP) at a concentration of 40 nm were applied to glow discharged 200 mesh Quantifoil grids (1.2µm hole size) by using a Vitrobot Mark IV (Thermo Fisher). The grids were blotted for 2 s in 100% humidity at 4 °C and were immediately plunged into liquid ethane. CryoEM images were collected at a nominal magnification of 79000 (pixel size of 1.5 Å) on an Glacios microscope (Thermo Scientific) operating at 200 kV and equipped with a Falcon4i Direct Electron Detector camera and Selectris X imaging filter. The defocus values were set to − 4 µm, the total dose was ≈ 16 electrons per Å^2^, and the energy filter was set to 10 eV. A total of ≈ 300 micrographs were acquired and the most representative were analyzed with the Velox software to measure the bacteriophages diameter.

### 2D Cell Culture

The human epidermal carcinoma cell line A431was grown in RPMI 1640 medium supplemented with 10% heat‐inactivated fetal bovine serum (FBS), 1% penicillin–streptomycin solution 100 U mL^−1,^ and 1% L‐glutamine 200 mm (Euroclone, Italy) at 37 °C in a humidified incubator with 5% CO_2_. Validation of M13_EGFR_(TNP) targeting on A431 cell line.

### Confocal Microscopy

A431 cells were seeded on round coverslips placed inside 6‐well plate (Corning) and grown overnight. Cells were then incubated for 45 min with a complete RPMI medium supplemented with either M13_EGFR_(TNP) or HSA‐TM bioconjugates at an equivalent concentration (1 nm). For analysis on their intracellular localization, MitoTracker™ Deep Red FM (Thermo Fisher Scientific) was added at the final concentration of 50 nm and incubated for 45 min. Cells were then washed thrice with PBS and stained for 15 min with Hoechst 33342 (Invitrogen) at the final concentration of 1 µg mL^−1^. A round coverslip was fitted into an Attofluor cell chamber (Invitrogen, USA) and covered with 1 mL of RPMI without phenol red supplemented with 10% FBS, 1% PenStrep, and 1% L‐glutamine. Cell images were then acquired with a NIKON A1R confocal microscope, maintaining a fixed laser setting during the acquisition of different samples. Semiquantitative analyses on the fluorescence intensity as well as on the fluorescence colocalization were performed with Fiji.^[^ ^104^ ^]^

### Flow Cytometry

The targeting of M13_EGFR_(TNP) and HSA‐TM bioconjugates to A431 cell line was evaluated by flow cytometry. M13_EGFR_(TNP) and HSA‐TM were incubated, at a final concentration of 1 nm, with 500000 adherent A431 cells for 45 min. Unbound agents were removed by washing thrice with PBS and cells were detached using trypsin 1x. After trypsin inactivation with RPMI complete, cells were washed twice, resuspended in 1 mL of PBS, and analysed with a CytoFLEX S (Beckam Coulter) cytofluorimeter. The fluorescence of at least 10000 events was evaluated in the PE channel (excitation λ = 488 nm, emission filter λ = 585/42nm). Data analysis was performed with CytExpert (Beckam Coulter) and FlowJo™.

### M13_EGFR_(TNP)‐Mediated Photodynamic Therapy

A431 cells, seeded in 96 well plates (Sarsted) were incubated for 45 min in the presence of 3D‐TNP, M13_EGFR_(TNP), and HSA‐TM at different concentrations. Afterward, cells were washed 3 times with PBS and irradiated for 10 min with LED light (24.0 mW cm^−2^ irradiance). Immediately after the treatment, PBS was removed, and cells were recovered 24 h in RPMI complete. Cell viability was evaluated through MTT assay and absorbance at 570 and 690 nm wavelength measured with EnSpire multimode plate reader (PerkinElmer, USA). Statistical analysis and IC_50_ calculations were performed using GraphPad Prism version 8.0 (GraphPad Software, California, USA). To investigate whether the photodynamic killing is associated with a regulated cell death mechanism, cells were detached with trypsin, incubated with Guava Nexin Reagent (Merck, Darmstadt, Germany), and analyzed through flow cytometry. The Guava Nexin Reagent contains 7‐aminoactinomycin (7‐AAD) and annexin V‐phycoerythrin fluorophores, which bind to permeable necrotic cells and to the surface exposed‐ phosphatidylserine of apoptotic cells, respectively. Conversely, live cells are not bound by either fluorophore. The percentage of apoptotic, necrotic and live cells was determined at 3 and 24 h after the PDT treatment.

### Intracellular ROS Production

The intracellular ROS production after PDT treatment was inferred by using ROS‐Glo™ H_2_O_2_ Assay (Promega) following the manufacturer's instructions. Briefly, ≈20000 A431 cells were incubated with M13_EGFR_(TNP) at different concentrations for 45 min, prior to washing and irradiation for 10 min with a LED light (24 mW cm^−2^ irradiance). Immediately after the treatment, 20 µL of H_2_O_2_ substrate solution were added to each well (final volume 100 µL) and incubated for 20 min at 37 °C. 100µL of ROS‐Glo™ detection solution were added to each sample and the plate was incubated at room temperature for 20 min. Relative luminescence was measured using EnSpire® multimode plate reader (PerkinElmer).

### Real‐Time Photokilling

Approximately 200000 A431 cells grown in adhesion on round coverslip were incubated for 45 min with RPMI complete supplemented with M13_EGFR_(TNP) at the final targeting agent concentration of 1 nm. Cells were washed to remove excess M13_EGFR_(TNP) and stained for 15 min with Hoechst 33342 (1 µg mL^−1^). Coverslips were fitted in an Attofluor cell chamber and covered with 1 mL of RPMI complete without phenol red. Confocal time lapses were obtained with a NIKON A1R confocal microscope and images were acquired every 2 min for 10 min irradiating with white light between each acquisition.

### Generation of 3D Spheroids

3D spheroids were generated through the hanging drop method.^[^ ^105^ ^]^ Briefly, adherent A431 cells were washed, trypsinized, and resuspended in RPMI complete (RPMI 1640 medium supplemented with 10% heat‐inactivated fetal bovine serum (FBS), 1% penicillin–streptomycin 100 U mL^−1^ and 1% L‐glutamine 200 mm). Next, a drop of 10 µL containing 1000 cells, was placed on the lid bottom of a 100 mm tissue culture dish. The lid was then inverted onto PBS‐filled culture dish and incubated at 37 °C with 5% CO_2_ for 5–7 days.

### Penetration of 3D‐TNP, M13_EGFR_(TNP) and M13(TNP) on 3D Spheroids

Homogeneous spheroids produced through hanging drop method were gently transfered to GravityTRAP Ultra‐Low Attachment 96 well plate (insphero) and incubated with RPMI complete supplemented with either 3D‐TNP, M13_EGFR_‐(TNP), M13(TNP) or HSA‐TM for 45 min. Spheroids were then washed thrice with PBS 1x, to remove unbound targeting agents, and stained for 15 min with Hoecsht 33342. Images of the bio‐conjugated targeting agents’ penetration into A431 spheroids were acquired every 45 min with NIKON A1R confocal microscope. Laser settings were maintained constant during the acquisition of the different samples. Fluorescence quantification was performed on the acquired images with Fiji.

### PDT of M13_EGFR_(TNP) on 3D Cellular Models

A431 spheroids transferred to GravityTRAP Ultra‐Low Attachment 96 well plate (insphero) and incubated with RPMI complete supplemented with either M13_EGFR_(TNP) or HSA‐TM for 45 min, were washed thrice with PBS and irradiated for 10 min with LED light (24 mW cm^−2^ irradiance). At the end of irradiation, PBS was removed, and 3D cell cultures were incubated with RPMI complete for 24 h at 37 °C with 5% CO_2_. Spheroid disaggregation was evaluated by the acquisition of images with NIKON A1R confocal microscope as previously described. Cell viability was evaluated by using CellTiter‐Glo® 3D Cell Viability Assay (Promega). Luminescence, which correlates with number of viable cells, was determined using EnSpire multimode plate reader (PerkinElmer).

### Animal Culture

*Hydra vulgaris* were cultured in Hydra medium (1 mm calcium chloride, 0.1 mm sodium hydrogen carbonate, pH 7). Animals were fed on alternate days with freshly hatched *Artemia nauplii* at 18 °C with a 12:12 h light: dark regime.

### Toxicological Evaluation of M13_EGFR_(TNP) in *Hydra vulgaris*

Toxicity studies in chronic conditions were performed to demonstrate the absence of toxicity of M13_EGFR_(TNP) and M13_EGFR_‐CF488A in absence of photostimulation in vivo. Groups of 10 animals were placed into a plastic multiwell wrapped by foil to simulate dark conditions. The animal morphology was monitored in response to increasing doses of M13_EGFR_(TNP) (0.01 to 1 nm) up to 72 h in dark conditions; every 24 h the polyps were washed with fresh Hydra solution and the treatment was repeated.

### Biodistribution of M13_EGFR_(TNP) in *Hydra vulgaris*

Groups of 5 polyps per condition were treated with 0.1 nm M13_EGFR_(TNP) for 30 min, 3 h and 24 h in a plastic multiwell covered by foil. After the incubation time, the polyps were washed several times with fresh Hydra solution and the images were acquired with an inverted microscope equipped with a Leica K5C digital microscope camera.

### In Vivo Photodynamic Treatment by using M13_EGFR_(TNP) in *Hydra vulgaris*

Groups of 10 polyps per condition were incubated with 0.1 nm M13_EGFR_(TNP) for 30 min in a plastic multiwell covered by foil. After the incubation, the animals were washed several times with fresh Hydra solution. A polyp at time was irradiated for 5, 15 or 30 min and the irradiation was performed with a mercury lamp filtered with a Zeiss filter (BP365/12–/FT395/LP397; light power density 0.04 mW cm^−2^).

### Gene Expression Analysis after the M13_EGFR_(TNP) Mediated Photodynamic Treatment in *Hydra Vvulgaris*

The expression levels of *SOD*, *Hsp70*, *Casp‐3*, and *Bcl‐2* were assessed by qRT‐PCR. Four conditions were analyzed: Untreated, Untreated irradiated, M13_EGFR_(TNP) treated and M13_EGFR_(TNP) treated and irradiated. For the irradiated conditions, a polyp per condition was irradiated as previously described for only 10 min as to induce a morphological mild damage and recovered in Hydra medium for 4 h. For each experimental condition, RNA was extracted from groups of 20 animals by purification in Trizol Reagent (Life Technologies) according to protocol. RNA was quantified and quality checked by SmartSpec plus spectrophotometer (Biorad, Hercules, CA) and agarose gel electrophoresis, respectively. RNA samples were treated with DNaseI (Amplification grade, Invitrogen) according to manufacturer's instructions. The first‐strand cDNA was synthesized by a High Capacity cDNA Reverse Transcription Kit (Applied Biosystem) using 0.5 µg of DNA‐free RNA in a final volume of 10 µL. qRT‐PCR was performed in 10 µL of reaction mixture consisting of 1x Express Sybr Green (Invitrogen), serial cDNA dilutions, and 0.5 µM each primer. The reactions were processed using the StepOne Real‐Time PCR System (Applied Biosystem) according to the following thermal profile: 50 °C for 2 min, one cycle for cDNA denaturation (94 °C for 2 min), followed by 40 amplification cycles (94 °C 2 s, 60 °C, 30 s). Specific pairs of primers were designed for each gene using the Primer3 program (http://frodo.wi.mit.edu) and are listed in Table S1 (Supporting Information). The expression profiles were analyzed by applying the ΔΔ*C*t method where the values of the gene of interest were normalized to the values of the reference control gene (*Ef1𝛼*).^[^ ^106^ ^]^

### Nematocyte Distribution in Tentacles

Nematocyte distribution was evaluated by toluidine‐blue staining. Polyps were relaxed for 1 min in 2% urethane in *Hydra* medium and fixed by pouring 99.5% ethanol. After 5 min polyps were rinsed several times in distilled water and stained with 0.05% toluidine blue in 10 mM Tris–HCl (pH 7.5). Following extensive washes polyps were dehydrated stepwise with 50, 75, and 95% ethanol and twice in 100% ethanol. Polyps were finally cleared in xylene and mounted in DPX, a non‐aqueous mounting medium. Stained tentacles were observed under bright field and phase‐contrast microscopy (Axiovert 100, Zeiss) equipped with a Leica K5C digital microscope camera.

## Conflict of Interest

The authors declare no conflict of interest.

## Supporting information



Supporting Information

## Data Availability

The data that support the findings of this study are available from the corresponding author upon reasonable request.
