# Supplementary material for: Phage‐Templated Synthesis of Targeted Photoactive 1D‐Thiophene Nanoparticles
Source: Small. 2024 Nov 5;21(1):2405832. doi: 10.1002/smll.202405832 (PMC11707577; doi:10.1002/smll.202405832)
Supplement: Supplementary file 1 — Supporting Information [file SMLL-21-2405832-s001.docx]

Supporting Information

**Phage-Templated Synthesis of Targeted Photoactive 1D-Thiophene Nanoparticles**

Paolo Emidio Costantini,^1,2†^ Roberto Saporetti,^3,†^ Marika Iencharelli,^4,†^ Soraia Flammini,^5^ Maria Montrone,^3^ Gennaro Sanità,^4^ Vittorio De Felice,^4^ Edoardo Jun Mattioli,^2,3^ Mattia Zangoli,^5^ Luca Ulfo,^1^ Michela Nigro,^1,2^ Tommaso Rossi,^1^ Matteo Di Giosia,^2,3^ Emanuela Esposito,^4^ Francesca Di Maria,^5^ Angela Tino^4^, Claudia Tortiglione,^4,*^ Alberto Danielli,^1,2*^ Matteo Calvaresi^2,3*^

*^1^ Dipartimento di Farmacia e Biotecnologie, Alma Mater Studiorum, Università di Bologna, Via Francesco Selmi 3, 40126 Bologna, Italy*

*^2^* *IRCCS Azienda Ospedaliero-Universitaria di Bologna, 40138 Bologna, Italy*

*^3^ Dipartimento di Chimica “Giacomo Ciamician, Alma Mater Studiorum, Università di Bologna, Via Francesco Selmi, 2, 40126 Bologna, Italy*

*^4^ Istituto di Scienze Applicate e Sistemi Intelligenti, Consiglio Nazionale delle Ricerche, Via Campi Flegrei 34, 80078 Pozzuoli, Italy.*

*^5^ Istituto per la Sintesi Organica e la Fotoreattività (ISOF), Consiglio Nazionale delle Ricerche, Via Piero Gobetti, 101, 40129 Bologna, Italy*

- 1. **Synthesis of the TM-like polymer**

The TM-like polymer (**P1**) was prepared following the general procedure for Stille Cross-coupling reported by us in [1], starting from 4,7-bis(5-bromo-3-hexylthiophen-2-yl)benzo[c][1,2,5]thiadiazole (**1**), synthesized according to [2] and 1,2-bis(5-(trimethylstannyl)thiophen-2-yl)ethene (**2**) (Figure S1). Two hexyl chains were added in TM to make the polymer more hydrophobic and facilitate the formation of the 3D-TNP in water.

The crude product was filtered, suspended in MeOH, and centrifuged three times. Deep red solid. Yield: 50%. ^1^H NMR (400 MHz, CDCl3, TMS/ppm): δ 7.68–6.69 (m), 3.16 (m), 2.44 (m), 1.27 (m), 0.90–0.76 (m).

**Figure S1.** Synthetic procedure for the synthesis of the TM-like polymer (P1).

**1.2 Synthesis of TM-like polymer nanoparticles (3D-TNP) and characterization**

As previously reported for similar thiophene-based polymers,^[3]^ TM-like polymer nanoparticles were synthesized *via* the reprecipitation method. A solution of the TM-like polymer (0.5 mg in 0.5 µL of THF) was added dropwise into 4 mL of Milli-Q water under constant stirring. Dynamic light scattering (DLS) analysis revealed that the nanoparticles had an average size of 95 ± 20 nm with a polydispersity index (PDI) of 0.184.


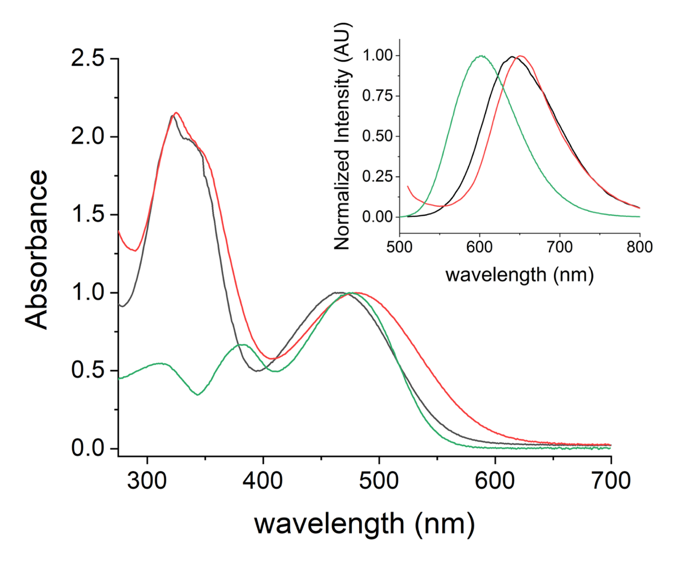


**Figure S2.** UV–visible spectra of 3D-TNP (red line) in water, TM-like polymer (black line) and NHS-TM (green line). The inset shows the fluorescence spectra of 3D-TNP (red line), TM-like polymer (black line) and NHS-TM (green line).

**
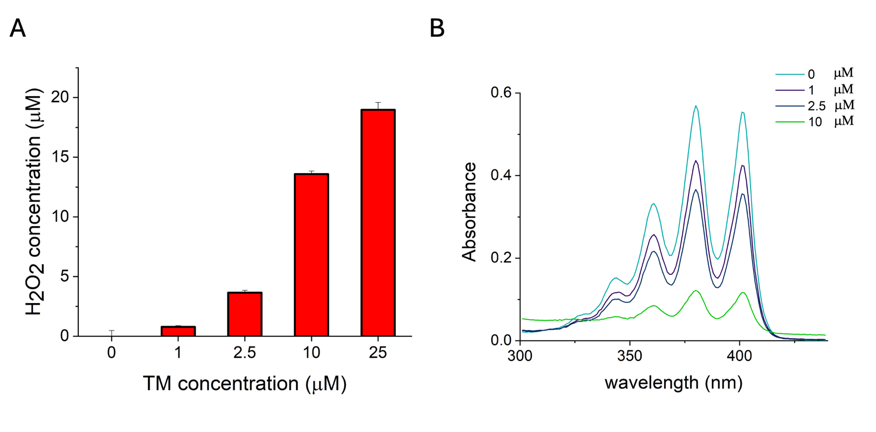
**

**Figure S3.** Photo-dependent ROS generation of 3D-TNP. **A**) Peroxide generation was estimated by measuring the fluorescence of resurfin, while **B**) singlet oxygen production was evaluated by measuring the decrease of the absorbance of the ABMDMA molecule.

**
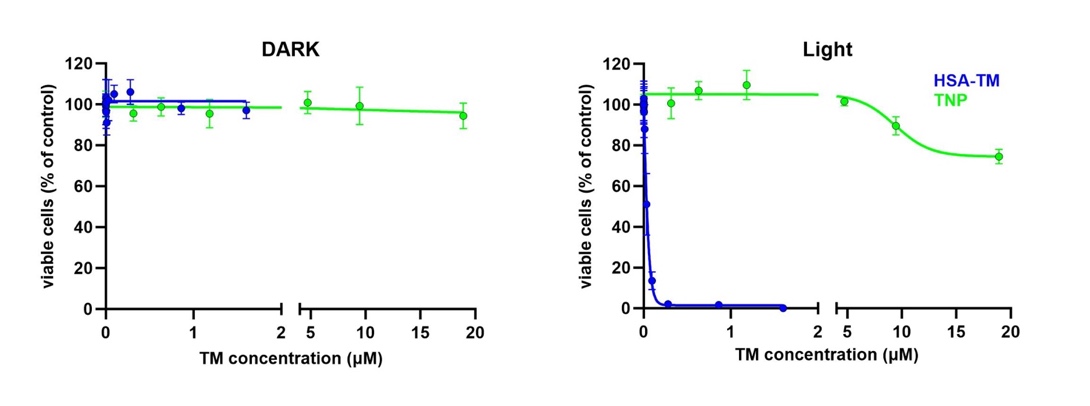
**

**Figure S4.** Photo-dependent cytotoxicity on cancer cells treated with HSA-TM or 3D-TNP. A431 cells incubated for 45 min with HSA-TM bioconjugates or 3D-TNP, were kept in dark condition or irradiated for 10 min with white light and cell viability was evaluated 24 h after the treatment. Data are shown as mean ± SD of 3 independent experiments and results are expressed as percentage of control (untreated - dark).

**
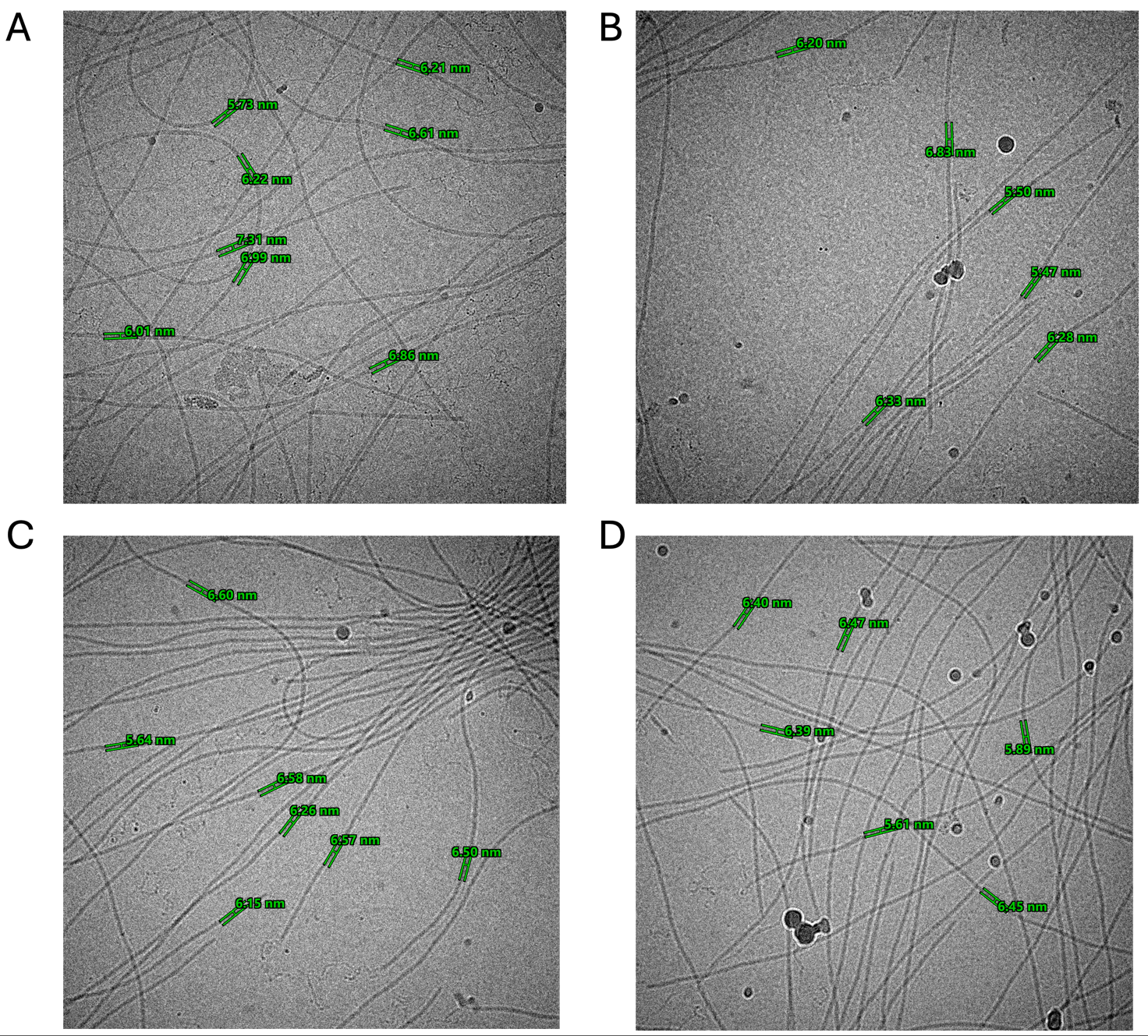
**

**Figure S5.** Cryo-TEM images of M13_EGFR_(TNP) in PBS 1x; the images are obtained from two different batches (A,B batch 1 – C,D batch 2).

**1.3.1 Effect of the amidation of NHS-TM**

To investigate the effect of the amidation reaction on the UV-vis spectrum of NHS-TM, we carried out the reaction in a simple model system, using ethyl diamine (EDA) in excess.

Two stock solutions were prepared:

NHS-TM 5mM in DMF

EDA 500mM in H_2_O (pH = 9.0) (EDA), using EDA dihydrochloride salt and NaOH 1M

2μL of NHS-TM stock solution was diluted with 448μL of DMF, then 50μL of EDA stock solution was added.

Considering the final concentration in solution of NHS-TM (20 μm) and EDA (50000 μm), a large excess of EDA is present (5000 reactive amine per each NHS-TM molecule).


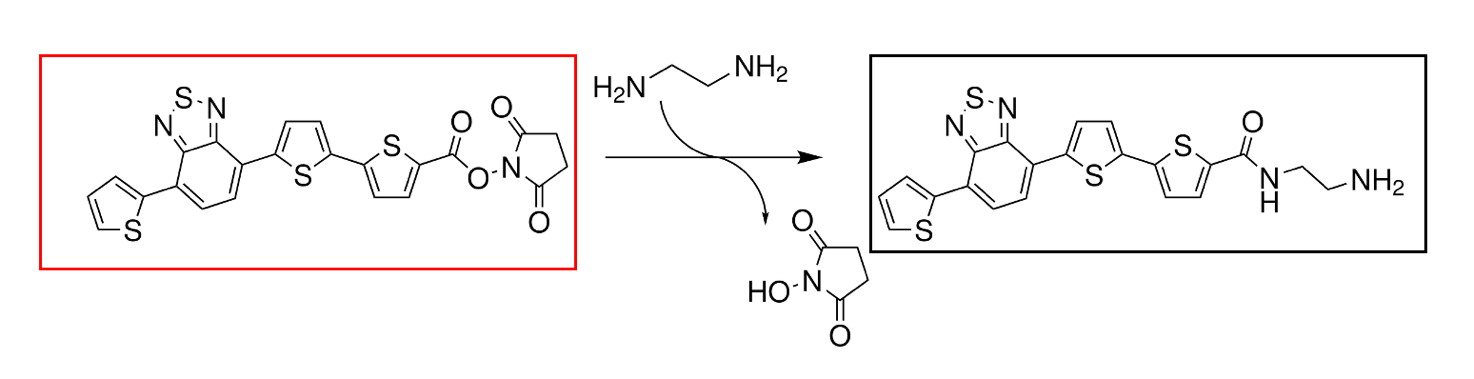


The UV-vis spectrum was recorded before and after addition of EDA. The reaction was quantitative (after 24h the UV-vis spectrum does not show variations).

**
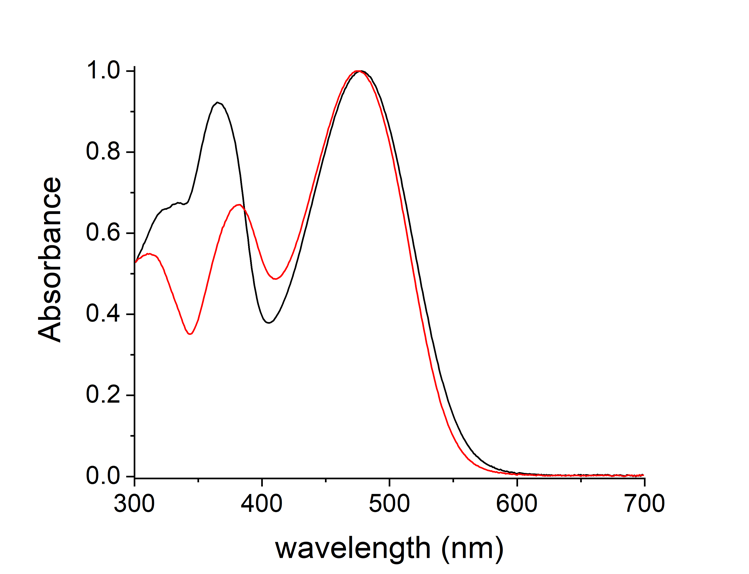
**

**Figure S6.** UV-vis spectra of NHS-TM, before (red line) and after (black line) amidation.

**1.3.2 TD-DFT Calculations on NHS-TM and amidated-TM**

TD-DFT calculations were carried out on NHS-TM and amidated-TM. All computations were carried out using Gaussian16 series of program.^[4]^ The geometry of the oligothiophenes molecules were optimized in gas phase, using the DFT functional CAM-B3LYP^[5]^ in conjunction with the 6-31+G* basis set (CAM-B3LYP/6-31+G*). UV-Vis spectra were calculated considering vertical transitions from the optimized ground state (S_0_) structure, using the Time-Dependent DFT formalism (TD-DFT) at the same level of theory (TD-CAM-B3LYP/6-31+G*). This combination well reproduces the photophysical properties of oligothiophene molecules.^[6]^

The calculated UV-Vis spectra of NHS-TM and amidated-TM well reproduce the obtained experimental result (Figure S7). S_0_->S_1_ transition is unaffected by the substituent group while S_0_->S_2_ transition shows a blue-shift of 11.4 nm and an increased oscillator strength when succinimide moiety is replaced with an amide group.


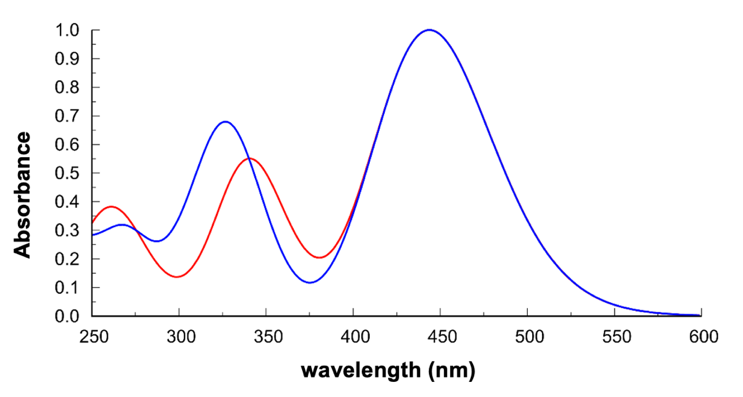


**Figure S7.** Calculated UV-vis spectra of NHS-TM (red line) and amidated-TM (blue line).

**
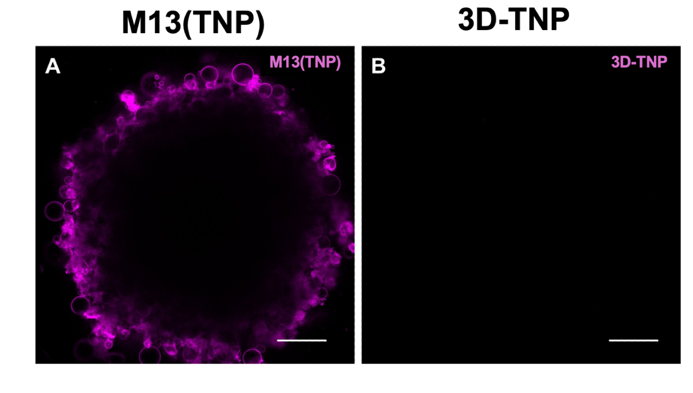
**

**Figure S8.** Targeting of M13(TNP) and 3D-TNP on 3D spheroids. Penetration into spheroid of (**A**) M13(TNP) and (**B**) 3D-TNP after 180’, using solutions of M13(TNP) and 3D-TNP isoabsorbing with M13_EGFR_(TNP). Fluorescence of the oligothiophene derivatives is shown in magenta. Scale bar = 100 µm.


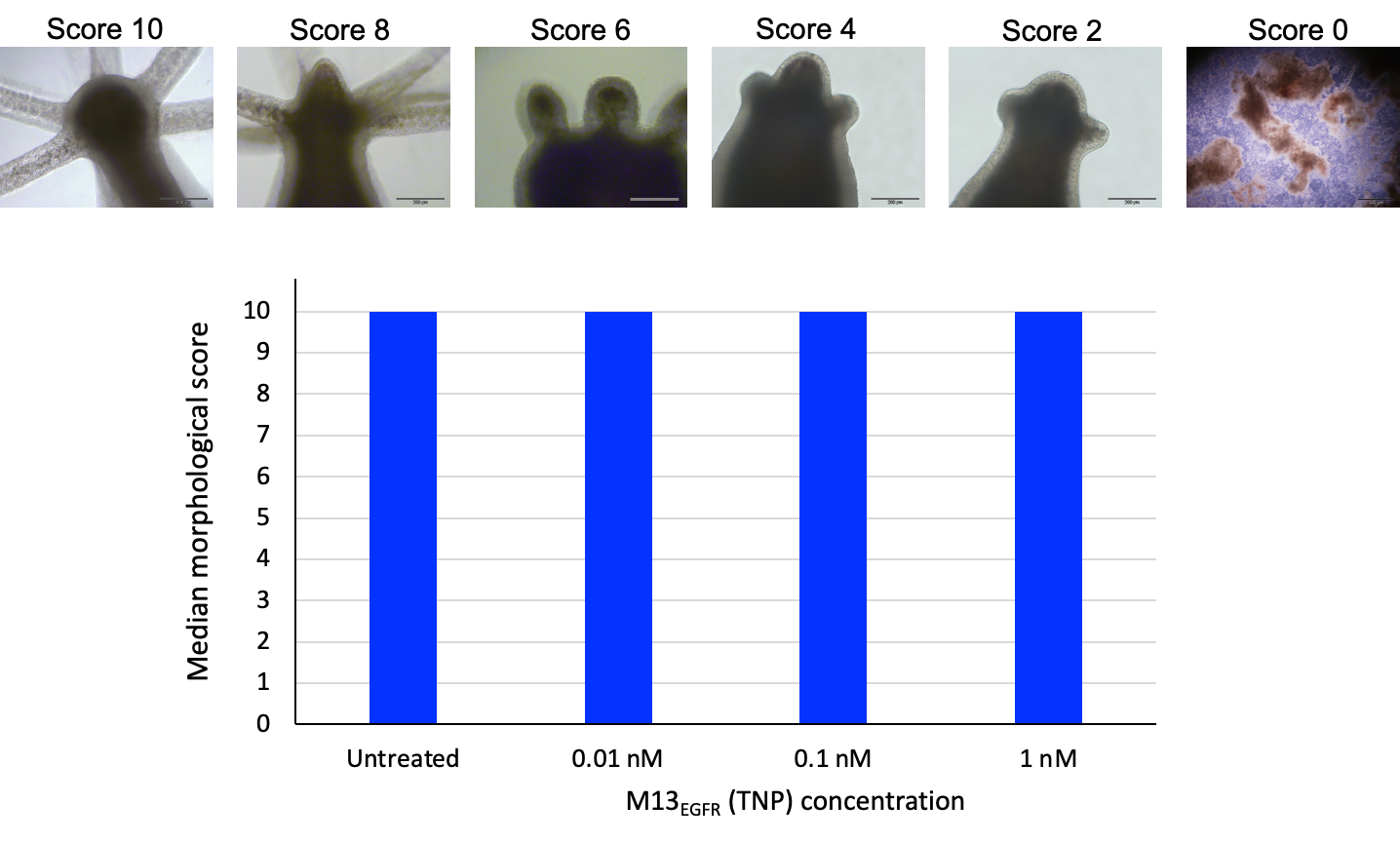


**Figure S9.** Toxicological evaluation of M13_EGFR_ (TNP) on *Hydra vulgaris*. Morphological changes and associated numerical score system employed for toxicological analysis. Upper panel: *Hydra* polyps respond to environmental stimuli and exogenous medium suspended compounds through a broad range of morphological changes, which range from tentacle contractions, then body contraction and swelling, up to tentacle loss and whole tissue disintegration. These morphological phenotypes can be quantified on a large number of animals by associating each phenotype to a numerical score, ranging from 10 (healthy animal) to zero (animal death). Lower panel: Dose−responses histograms of *Hydra* polyps exposed to different doses of M13 _EGFR_ (TNP). All treated polyps appeared healthy, with a median score equal to 10. Three independent experiments were carried out (n=30).


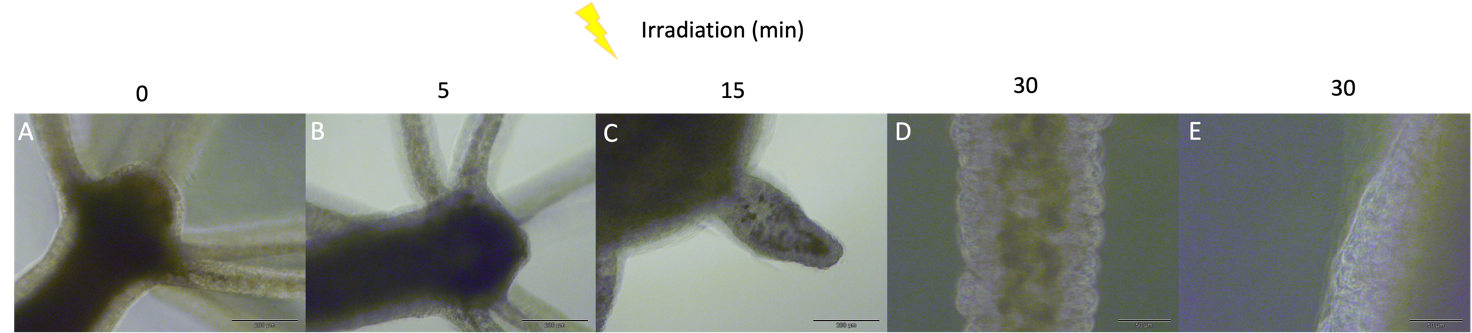


**Figure S10.** Biosafety of the NIR irradiation on *Hydra* tissue Living polyps were irradiated for the indicated periods with a mercury lamp filtered with a Zeiss filter (BP365/12– FT395 – LP397; light power density 0.04 mW cm^-2^). The images show the absence of cell and tissue damages, confirming the biosafety of the irradiation. Scale bars 200 μm A, B, C and 50 μm D, E.

**Figure S11. Toxicological evaluation of M13_EGFR_-CF488A in *Hydra vulgaris***

Polyps were continuously incubated h with M13_EGFR_-CF488A at the indicated concentrations up to 72 h. Data show biosafety in every condition at every time point. n=10. Scale bars: 500 μm.

**
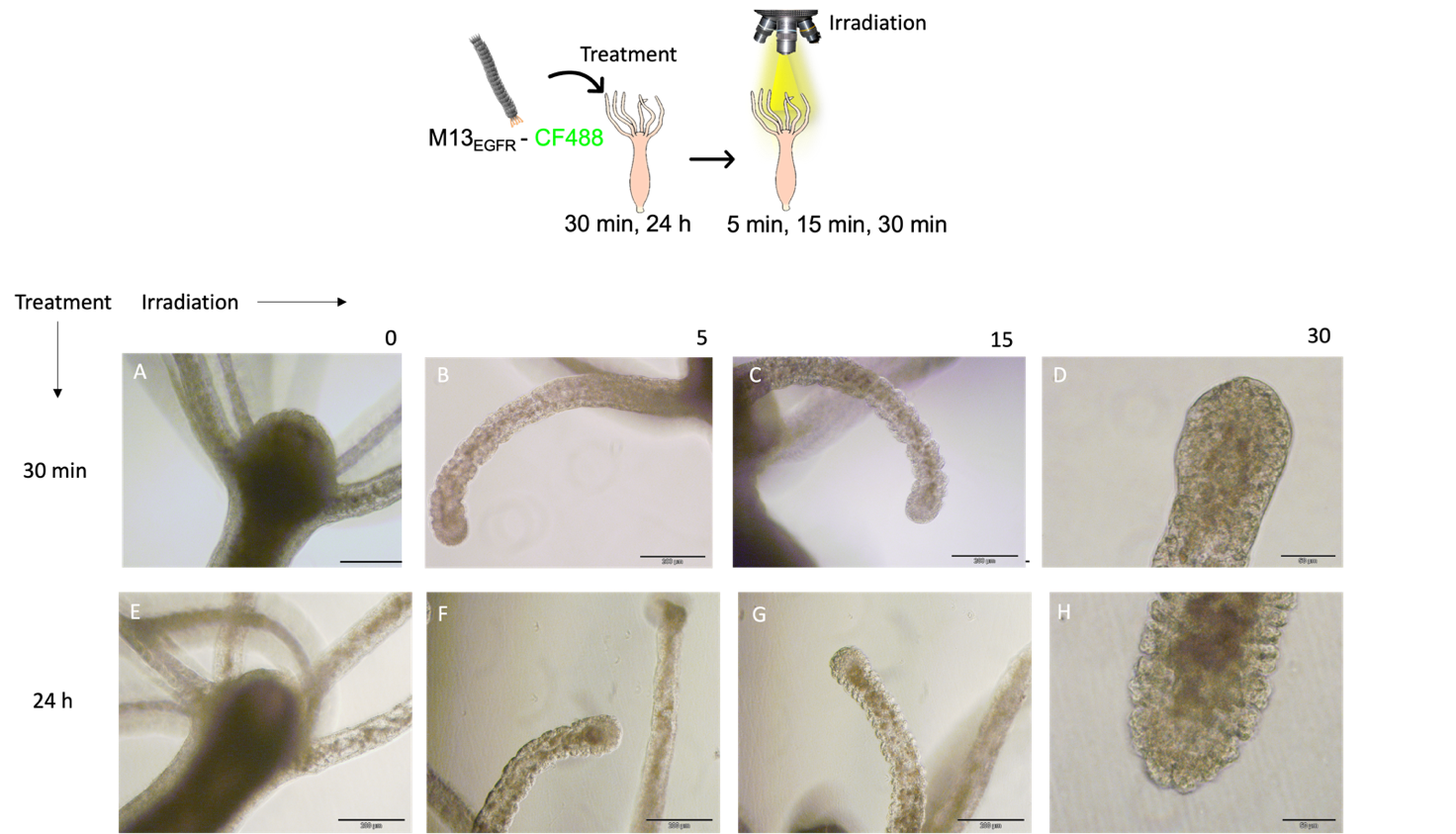
**

**Figure S12.** M13_EGFR_-CF488A engineered phage does not induce cell ablation in *Hydra.* Polyps were treated with 0.1 nM M13_EGFR_-CF488A for 30 min and 24 h and irradiated for the indicated periods. Irradiation was performed with a mercury lamp filtered with a Zeiss filter (BP365/12– FT395 – LP397; light power density 0.04 mW cm^-2^). The images show the absence of cell and tissue damages in any experimental condition. Scale bars 200 μm A, B, C, E, F, G and 50 μm D, H.

**Table S1. List of forward and reverse primers employed in the qRT-PCR analysis.**

| Gene | accession number | Forward primer | reverse primer | amplicon length (bp) |
| --- | --- | --- | --- | --- |
| *HySOD* | XP_002162688 | agactcccaaattcccttgg | ccaatgacaccacaggacaa | 149 |
| *HyCASP3* | XP_002165630.1 | gatggacctggaaatcttgc | cgcatattgacgaaaaactcc | 167 |
| *HyEf-1a* | Z68181.1 | ccaggagacaatgtcggttt | gcttcaatggcaggatcatt | 157 |
| *Hsp70.1* | XP_002159813 | cgacgtattcagacaatcaacc | caatttgaggaacacctcttgg | 136 |
| *Bcl-2 like 4* | XP_002167578 | aacaaggtggatgggatgg | ataagtaatgcgcccacacc | 147 |

References

[1] T. Moreira, F. Di Maria, M. Zangoli, E. Fabiano, I. Manet, R. Mazzaro, V. Morandi, M. Marinelli, G. Gigli, A. J. Parola, C. A. T. Laia, Giovanna Barbarella *Adv. Electron. Mater*. **2021**, 7, 2100166

[2] M. Zangoli, F. Monti, A. Zanelli, M. Marinelli, S. Flammini, N. Spallacci, A. Zakrzewska, M. Lanzi, E. Salatelli, F. Pierini, F. Di Maria *Chem. Eur. J.,* **2024**, 30, e202303590

[3] M. Zangoli, F. Di Maria, *View* **2020**, *2*, 20200086.

[4] Gaussian 16, Revision C.01, M. J. Frisch, G. W. Trucks, H. B. Schlegel, G. E. Scuseria, M. A. Robb, J. R. Cheeseman, G. Scalmani, V. Barone, G. A. Petersson, H. Nakatsuji, X. Li, M. Caricato, A. V. Marenich, J. Bloino, B. G. Janesko, R. Gomperts, B. Mennucci, H. P. Hratchian, J. V. Ortiz, A. F. Izmaylov, J. L. Sonnenberg, D. Williams-Young, F. Ding, F. Lipparini, F. Egidi, J. Goings, B. Peng, A. Petrone, T. Henderson, D. Ranasinghe, V. G. Zakrzewski, J. Gao, N. Rega, G. Zheng, W. Liang, M. Hada, M. Ehara, K. Toyota, R. Fukuda, J. Hasegawa, M. Ishida, T. Nakajima, Y. Honda, O. Kitao, H. Nakai, T. Vreven, K. Throssell, J. A. Montgomery, Jr., J. E. Peralta, F. Ogliaro, M. J. Bearpark, J. J. Heyd, E. N. Brothers, K. N. Kudin, V. N. Staroverov, T. A. Keith, R. Kobayashi, J. Normand, K. Raghavachari, A. P. Rendell, J. C. Burant, S. S. Iyengar, J. Tomasi, M. Cossi, J. M. Millam, M. Klene, C. Adamo, R. Cammi, J. W. Ochterski, R. L. Martin, K. Morokuma, O. Farkas, J. B. Foresman, and D. J. Fox, Gaussian, Inc., Wallingford CT, **2016**.

[5] T. Yanai, D.P. Tew, N.C Handy *Chemical Physics Letters* **2004,** *393*, 51-57.

[6] A. Cantelli, M. Malferrari, A. Soldà, G. Simonetti, S. Forni, E. Toscanella, E.J. Mattioli, F. Zerbetto, A. Zanelli, M. Di Giosia, M. Zangoli, G. Barbarella, S. Rapino, F. Di Maria, and M. Calvaresi *JACS Au* **2021** *1*, 925-935.
